# Environmental and Ecological Monitoring with Biodegradable Technologies

**DOI:** 10.1002/advs.202511452

**Published:** 2025-12-01

**Authors:** Mohammad Javad Bathaei, Yaren Bathaei, Zhengwei Liao, Maryam Yazdanmehr, Sarab S. Sethi, Denys Nikolayev, Filipe Arroyo Cardoso, Clementine M. Boutry

**Affiliations:** ^1^ Department of Microelectronics Delft University of Technology 2628 CD Delft The Netherlands; ^2^ Department of Aerospace Structures and Materials Delft University of Technology 2629 HS Delft The Netherlands; ^3^ Department of Life Sciences Imperial College London London W12 0BZ UK; ^4^ Institut d’électronique et des Technologies du numérique (IETR UMR 6164) French National Center for Scientific Research (CNRS) / University of Rennes Rennes 35042 France

**Keywords:** ecological monitoring, environmental monitoring, sustainable device, sustainable powering, transient electronics

## Abstract

The emergence of a new family of wireless biodegradable sensors marks a groundbreaking leap in ecological and environmental sensing. These biodegradable devices can collect a wide range of data in agriculture, climate research, forestry, water management, and biodiversity protection. Manufactured primarily from environmentally safe transient materials for sensing and data transmission, these systems undergo controlled degradation after use, minimizing environmental electronic waste. Here, a critical review of key aspects in the development and application of biodegradable sensors is performed for ecological and environmental monitoring. First, the different materials utilized in the development of biodegradable environmental monitoring devices and their applications are explored. The relevant degradation mechanisms, including hydrolysis, oxidation, photodegradation, and micro‐organism action are examined as a function of environmental conditions. Then compatible and non‐toxic fabrication techniques are investigated for building biodegradable sensors, emphasizing their scalability and potential for mass production. Finally, system‐level considerations are discussed for sustainable powering of these devices, ensuring efficient operation while maintaining environmental sustainability. By surveying a broad spectrum of applications and ongoing advancements, it is argued that biodegradable sensors have a transformative potential in advancing sustainable, widespread, and cost‐effective ecological and environmental monitoring solutions.

## Graphical abstract

This review examines the development and application of wireless biodegradable sensors for environmental monitoring. It explores (bio)degradable materials, their degradation mechanisms in various environments, and non‐toxic fabrication techniques. Additionally, it addresses scalable production and sustainable powering solutions, emphasizing the high potential of these sensors for advancing sustainable and cost‐effective ecological monitoring across diverse fields.

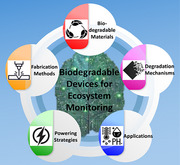

## 1 Introduction

Advancements in environmental monitoring technologies—such as satellites,^[^ ^1^ ^]^ camera traps,^[^ ^2^ ^]^ acoustic recorders,^[^ ^3^ ^]^ radio‐frequency identification (RFID) tags,^[^ ^4^ ^]^ and environmental sensors measuring temperature,^[^ ^5^ ^]^ humidity,^[^ ^6^ ^]^ pH,^[^ ^7^ ^]^ and pollutant levels^[^ ^8^ ^]^—have significantly enhanced our capacity to understand the local and global evolution of the climate and ecological dynamics. These innovations enable the development of smarter approaches to agriculture and biodiversity protection.^[^ ^9^ ^]^

Regarding ecology monitoring, satellites are critical for large‐scale biodiversity assessments and landscape‐level monitoring, though their resolution is often insufficient for detecting fine‐scale, species‐specific patterns.^[^ ^10^ ^]^ Camera traps provide detailed insights into the presence, abundance, and behavior of terrestrial animals; however, their physical presence can disrupt natural habitats, and their deployment is limited to specific areas.^[^ ^2^, ^11^ ^]^ Acoustic recorders enable the assessment of animal communities, particularly vocal species. Yet, their utility can be compromised by ambient noise and their inability to distinguish non‐vocal species.^[^ ^12^ ^]^ RFID technology allows for precise tracking of individual animal movements but often necessitates invasive tagging methods, potentially altering animal behavior and ecosystem interactions.^[^ ^4^ ^]^ Similarly, sensors that measure physical and chemical parameters, such as temperature, pH, and contaminant levels, provide critical environmental data for both ecological and agricultural applications. Depending on the specific application context, life‐cycle assessments (LCAs) have shown that biodegradable devices can offer environmental advantages over non‐biodegradable systems, particularly in short‐term monitoring, single‐use deployments, or operations in sensitive ecosystems where device retrieval is impractical and long‐term residue must be minimized.^[^ ^5^, ^6^, ^7^, ^8^, ^13^, ^14^ ^]^

To overcome these challenges, the emergence of ecoresorbable and biodegradable electronics presents a sustainable and innovative solution.^[^ ^15^ ^]^ According to the commonly accepted definition, ecoresorbable devices, which degrade into soluble constituents, can significantly reduce the generation of solid waste.^[^ ^16^ ^]^ Biodegradable devices gradually break down over time into environmentally safe materials through biological processes, such as the activity of microorganisms, enzymes, and other natural mechanisms. However, the environment may not fully absorb them.^[^ ^17^ ^]^ The use of ecoresorbable/biodegradable materials can reduce the environmental impact and prevents further contributions to electronic waste (e‐waste).^[^ ^18^ ^]^ They address critical challenges such as ecotoxicity and the retrieval of non‐biodegradable devices post‐deployment.^[^ ^19^, ^20^ ^]^ Moreover, ecoresorbable/biodegradable devices offer a significant advantage in scalability for measurements, as they can be mass‐produced and widely distributed to collect vast amounts of data while naturally decomposing.^[^ ^17^, ^21^, ^22^, ^23^, ^24^ ^]^ This promising technology mitigates environmental harm and enhances the potential scale and sustainability of monitoring systems, marking a significant advancement in the field.^[^ ^25^ ^]^

**Figure 1:**
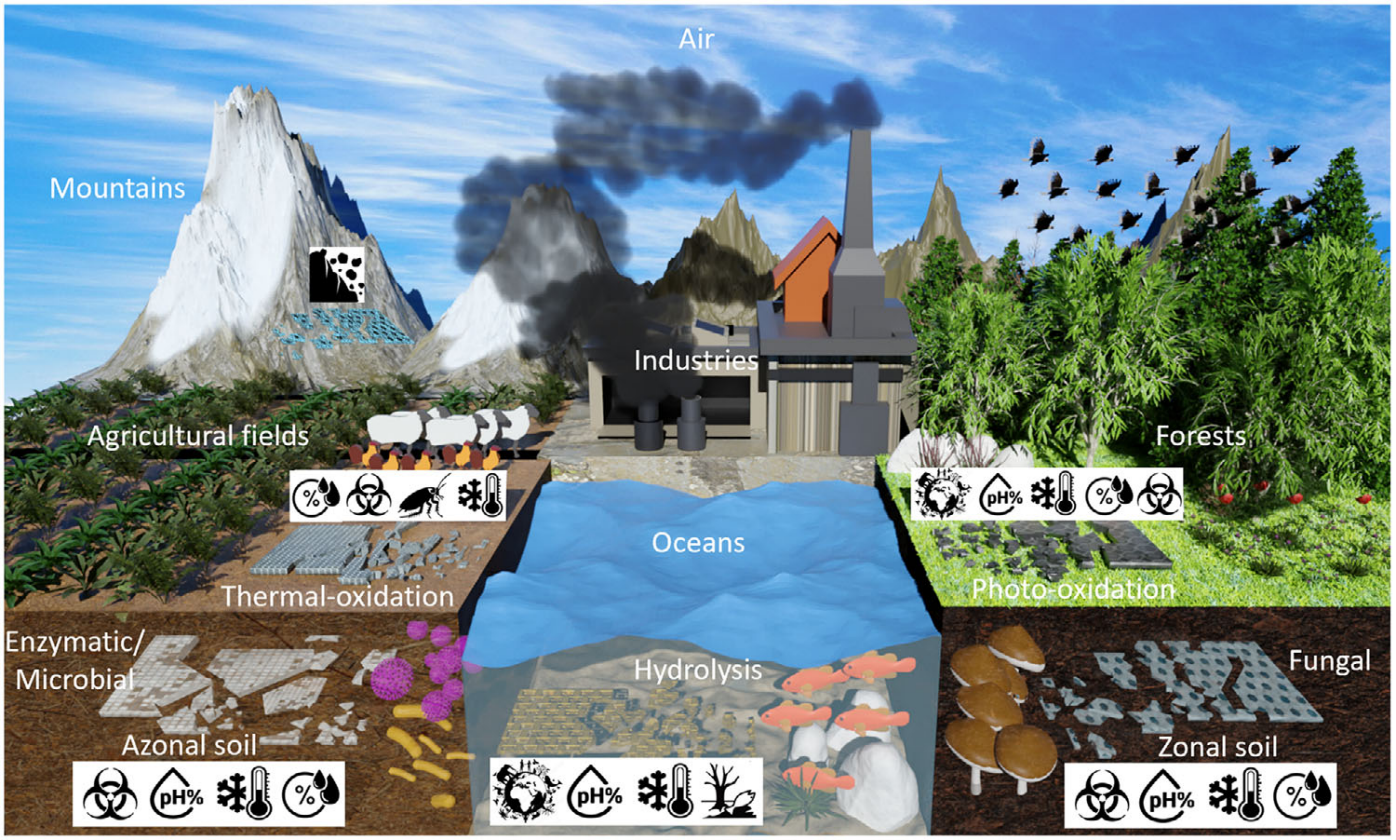
Conceptual illustration of biodegradable devices for monitoring humidity, temperature, pH, pesticides, biodiversity, chemicals, and geological events across ecosystems such as agricultural fields, air, oceans, forests, soils, and mountains. Devices undergo environment‐specific degradation mechanisms, including hydrolysis, enzymatic activity, fungal degradation, and photo/thermal oxidation.

The earliest partially/fully biodegradable environmental monitoring devices, made from substrates like paper,^[^ ^26^, ^27^ ^]^ silk,^[^ ^28^ ^]^ and wheat gluten proteins,^[^ ^29^ ^]^ incorporated simple, non‐biodegradable resistive or interdigital capacitive electrodes and served as passive sensors for humidity, temperature, and gas detection. With advancements in this field, these sensors have been adapted for various environmental and ecological applications. **Figure** **1** shows their applications in environmental and ecological monitoring.

Designed to degrade according to their environmental conditions, ecoresorbable/biodegradable sensors utilize tailored mechanisms to ensure minimal ecological impact. Depending on factors like moisture, microbial activity, and sunlight exposure, these devices break down via hydrolysis,^[^ ^30^ ^]^ oxidation,^[^ ^31^ ^]^ enzymatic action,^[^ ^32^, ^33^ ^]^ fungal degradation,^[^ ^34^ ^]^ or photodegradation.^[^ ^35^ ^]^ Such diverse degradation pathways highlight the adaptability and sustainability of transient electronics in environmental/ecological applications.

Ecoresorbable/biodegradable devices represent emerging sustainable solutions for monitoring various physical, chemical, and biological processes across diverse application domains.^[^ ^36^, ^37^, ^38^ ^]^ Ongoing research in agriculture has demonstrated the potential of ecoresorbable and biodegradable sensors for assessing soil health parameters such as nutrient content, moisture, and microbial activity to enhance crop productivity and support sustainable farming practices.^[^ ^39^, ^40^, ^41^ ^]^ In forestry studies, prototype systems have been explored for monitoring soil moisture and humidity, while in aquaculture, experimental pH sensors have shown promise in maintaining optimal water conditions for aquatic organisms.^[^ ^42^ ^]^ Similarly, in wetlands and natural water bodies, biodegradable sensors have been tested for tracking pH fluctuations to support ecosystem protection, decomposing naturally to avoid disrupting sensitive habitats.^[^ ^30^, ^43^, ^44^, ^45^, ^46^, ^47^ ^]^ Preliminary work has also examined their use in climate and atmospheric studies, as well as in monitoring pH levels in industrial runoff and wastewater treatment to mitigate environmental contamination and support regulatory compliance.^[^ ^21^, ^48^ ^]^

Beyond pH monitoring, transient sensors play a crucial role in real‐time chemical detection, providing immediate data on pollutants in air and water. This enables prompt action to mitigate pollution and supports environmental conservation efforts.^[^ ^49^, ^50^ ^]^ Temperature sensors further expand their utility by tracking fluctuations across ecosystems, aiding in climate change studies, monitoring sensitive environments, and ensuring regulatory compliance in industries.^[^ ^45^, ^51^ ^]^ Geological applications can potentially benefit from biodegradable sensors, particularly in landslide detection. For instance, wireless sensor network‐based devices like SMARTMODE have been designed to efficiently monitor landslide movements while adapting to environmental changes and optimizing energy usage.^[^ ^46^, ^52^ ^]^

This review begins with the exploration of biodegradable materials utilized in the fabrication of transient devices for environmental/ecological monitoring. We provide insights into the categorization and application of these materials across functional and supportive layers within environmental/ecological monitoring devices. We further examine the diverse degradation mechanisms and key limitations of current sensors, followed by a discussion of the broad spectrum of applications for biodegradable technologies. We also discuss system‐level integration, including the role of sensor hubs, hybrid interfacing with conventional silicon‐based electronics, communication architectures, and end‐of‐life management strategies, outlining practical implementation pathways and future research directions. Finally, we explore various fabrication techniques and powering methods tailored for these devices, highlighting ongoing advancements aimed at improving reliability, efficiency, and scalability.

## 2 Methods

A systematic literature search was conducted using the Web of Science Core Collection to identify research on partially/fully biodegradable devices developed for environmental and ecological monitoring applications. The search covered the period 2014–2024, included only English‐language journal articles, and was performed in both Title (TI) and Abstract (AB) fields to ensure comprehensive coverage.

Search strings combined biodegradability‐related terms (biodegradable, degradable, ecoresorbable, eco‐friendly, transient, green) with sensor‐ and environment‐related descriptors (sensor, device, electronic, environmental, ecological, agricultural, climatic, sustainability). Additional keyword sets targeted specific sensor categories, including moisture/humidity, pH, chemical (including gas), temperature, and photodetector/optical sensors.

The truncation symbol (*) was used to capture all word variations (e.g., ecofriend* → ecofriendly, eco‐friendliness; degrad* → degradable, degradation), ensuring comprehensive coverage. The detailed search results and the number of papers found and included are summarized in **Table** **1**.

**Table 1:** Systematic literature search strategy for partially/fully biodegradable sensors for environmental and ecological monitoring.

Sensor type	Search field	Search keywords	Papers found	Papers included in review
Moisture / Humidity	TI	((biodegradable OR degradable OR transient OR ecoresorbable OR ecofriend* OR compost* OR decompos* OR dispos*) AND (sensor* OR device* OR patch OR electronic*) AND (ecolog* OR environment* OR ecosystem* OR agricultur* OR farm* OR cultivat* OR climate OR weather) AND (moisture OR humid* OR fog* OR water OR wet* OR mist* OR rain*))	1	1
	AB	((biodegradable OR degradable OR transient OR ecoresorbable OR ecofriend* OR compost* OR decompos* OR dispos*) AND (sensor* OR device* OR patch OR electronic*) AND (ecolog* OR environment* OR ecosystem* OR agricultur* OR farm* OR cultivat* OR climate OR weather) AND (moisture OR humid* OR fog* OR water OR wet* OR mist* OR rain*))	1720	34
pH	TI	((biodegradable OR degradable OR transient OR ecoresorbable OR ecofriend* OR compost* OR decompos* OR dispos*) AND (sensor* OR device* OR patch OR electronic*) AND (ecolog* OR environment* OR ecosystem* OR agricultur* OR farm* OR cultivat* OR climate OR weather) AND (pH OR alkali* OR base* OR acid* OR hydrogen*))	10	1
	AB	((biodegradable OR degradable OR transient OR ecoresorbable OR ecofriend* OR compost* OR decompos* OR dispos*) AND (sensor* OR device* OR patch* OR electronic*) AND (ecolog* OR environment* OR ecosystem* OR agricultur* OR farm* OR cultivat* OR climate OR weather) AND (pH OR alkali* OR base* OR acid*))	2832	9
Chemical	TI	((biodegradable OR degradable OR transient OR ecoresorbable OR ecofriend* OR compost* OR decompos* OR dispos*) AND (sensor* OR device* OR patch* OR electronic*) AND (ecolog* OR environment* OR ecosystem* OR agricultur* OR farm* OR cultivat* OR climate OR weather) AND (chemical* OR gas* OR compound* OR analyte* OR pollut* OR contaminat* OR substance* OR nitrate OR inorganic* OR organic*))	17	5
	AB	((biodegradable OR degradable OR transient OR ecoresorbable OR ecofriend* OR compost* OR decompos* OR dispos*) AND (sensor* OR device* OR patch* OR electronic*) AND (ecolog* OR environment* OR ecosystem* OR agricultur* OR farm* OR cultivat* OR climate OR weather) AND (chemical* OR gas* OR compound* OR analyte* OR pollut* OR contaminat* OR nitrate* OR inorganic* OR organic*))	3619	26
Temperature	TI	((biodegradable OR degradable OR transient OR ecoresorbable OR ecofriend* OR compost* OR decompos* OR dispos*) AND (sensor* OR device* OR patch* OR electronic*) AND (ecolog* OR environment* OR ecosystem* OR agricultur* OR farm* OR cultivat* OR climate OR weather) AND (temperature* OR heat OR warm* OR therm* OR cold))	9	1
	AB	((biodegradable OR degradable OR transient OR ecoresorbable OR ecofriend* OR compost* OR decompos* OR dispos*) AND (sensor* OR device* OR patch* OR electronic*) AND (ecolog* OR environment* OR ecosystem* OR agricultur* OR farm* OR cultivat* OR climate OR weather) AND (temperature* OR heat OR warm* OR therm* OR cold))	2238	29
Photodetectors	TI	((biodegradable OR degradable OR transient OR ecoresorbable OR ecofriend* OR compost* OR decompos* OR dispos*) AND (photodetector* OR photosensor* OR optical sensor* OR light sensor* OR photoresistor* OR photodiode*) AND (ecolog* OR environment* OR ecosystem* OR agricultur* OR habitat OR climate OR weather))	8	2
	AB	((biodegradable OR degradable OR transient OR ecoresorbable OR ecofriend* OR compost* OR decompos* OR dispos*) AND (photodetector* OR photosensor* OR optical sensor* OR light sensor* OR photoresistor* OR photodiode*) AND (ecolog* OR environment* OR ecosystem* OR agricultur* OR habitat OR climate OR weather))	1145	4

The systematic search primarily focused on identifying functional biodegradable sensors experimentally demonstrated in environmental or ecological contexts. The resulting publications formed the foundation of this review. Based on the experimental results, methodologies, and analyses reported in these studies, additional sections were developed to synthesize insights on: i) materials employed for different functional components of the devices—such as substrates, electrodes, sensing layers, and encapsulation—to achieve both device functionality and environmental degradability; ii) degradation mechanisms associated with these materials and their interaction with the surrounding environment, influencing device stability, performance, and end‐of‐life disintegration; iii) fabrication techniques implemented in the reviewed studies, as well as scalable, green, and sustainable approaches promising for future device manufacturing; and iv) powering and energy‐harvesting strategies, encompassing both technologies already demonstrated and emerging eco‐efficient solutions that enable autonomous, self‐sustained sensor operation.

These thematic sections were derived from the content of the selected studies, ensuring a cohesive, application‐oriented overview of current advances and future directions in biodegradable environmental sensing technologies.

## 3 Biodegradable Materials Selection

The development of transient devices for environmental and ecosystem monitoring hinges fundamentally on the selection of materials to ensure both functionality and biodegradability. Functional components require materials with specific electrical, chemical, mechanical, magnetic, and optical properties.^[^ ^30^, ^42^, ^53^, ^54^, ^55^, ^56^, ^57^, ^58^ ^]^ In wireless ecoresorbable devices, the thickness of conductive materials influences conductance and degradation rate; higher conductivity improves electrical performance, with minor skin‐effect at high frequencies, while greater thickness reduces resistance but slows biodegradation.^[^ ^32^ ^]^ Simultaneously, the mechanical strength and flexibility of structural materials influence durability and environmental resilience, while their biodegradation rate determines the device's end of life. Thus, selecting materials tailored to specific applications is essential for successfully developing and deploying biodegradable devices.^[^ ^59^, ^60^, ^61^, ^62^, ^63^ ^]^

Previous studies, notably recent reviews by Shim et al.,^[^ ^64^ ^]^ Ryu et al.,^[^ ^65^ ^]^ Singh et al.,^[^ ^66^ ^]^ and Zhang et al.^[^ ^67^ ^]^ provide a comprehensive overview of biodegradable materials used in transient sensors, detailing their mechanical, electrical, chemical, and degradation properties. As this information has been covered extensively, the present review will instead focus on common materials used in sensors for environmental and ecosystem monitoring, as summarized in **Table** **2**.

**Table 2:** Common materials and related degradation mechanisms in environmental monitoring devices.

Components	Materials	Type		Degradation mechanism	Refs.
Substrate	Cellulose	Natural polymer		Fungal/Bacterial	[43, 81, 82]
	Wood	Natural composite		Fungal/Bacterial	[83, 84]
	Chitosan	Natural polymer	Enzymatic/Photo‐oxidation		[77, 85]
	Silk	Natural polymer	Enzymatic/Fungal		[86]
	PVA	Synthetic polymer	Hydrolysis/Thermal oxidation		[77]
	PPLA	Synthetic polymer	Bacterial/Photo‐oxidation		[32, 35]
Encapsulation	Ecoflex (corn starch, potato, and PLA)	Composite	Hydrolysis		[42]
	Polyanhydrides	Synthetic polymer	Hydrolysis		[87]
	Bees/soy wax	Natural composite	Hydrolysis		[88]
	Bees/konjac wax	Natural composite	Hydrolysis		[89, 90]
Conductor	Mg/Fe	Inorganic	Hydrolysis		[77, 91]
	Zn	Inorganic	Hydrolysis		[32]
	Mo	Inorganic	Hydrolysis		[92]
	Mg	Inorganic	Hydrolysis		[92]
	W	Inorganic	Hydrolysis		[93]
	Graphene/Reduced graphene oxide (rGO)	Inorganic	Bacterial/Oxidation		[94, 95]
Semiconductor	Zinc oxide	Inorganic	Hydrolysis		[48]
	Si nanomembrane	Inorganic	Hydrolysis		[42]
	MgO	Inorganic	Hydrolysis		[96]
	MoO_3_	Inorganic	Hydrolysis		[97]
Dielectric	SiO_2_	Inorganic	Hydrolysis		[42]
	Si_3_N_4_	Inorganic	Hydrolysis		[42]
	Egg albumen	Natural polymer	Bacterial		[43, 89]
	Gelatin	Natural polymer	Fungal/Bacterial		[98, 99]
	Casein	Natural polymer	Fungal/Bacterial		[100, 101]

**Figure 2:**
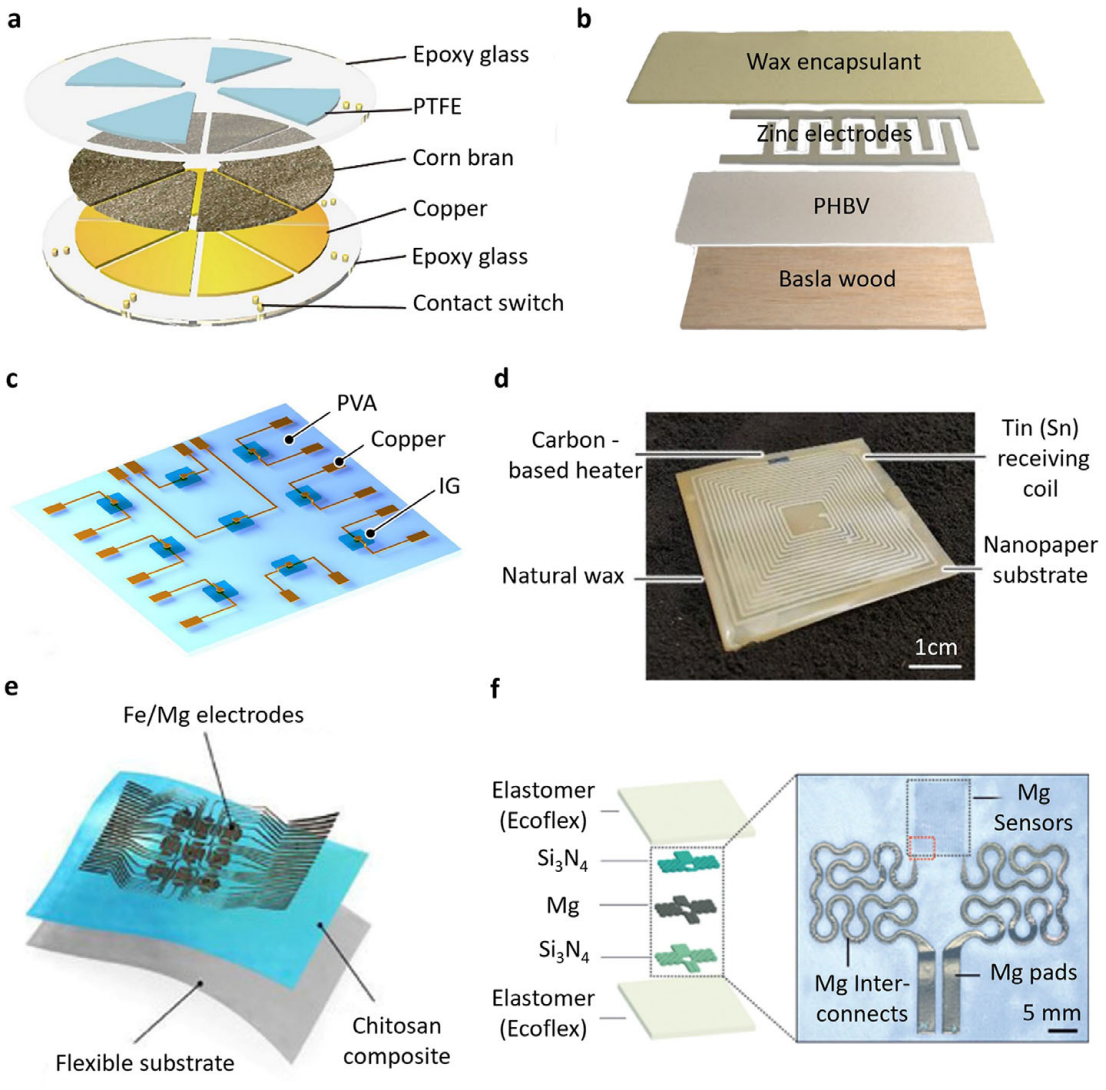
Various transient materials used in environmental monitoring technologies: a) Schematic of triboelectric nanogenerator (TENG) utilizing corn bran as a power source for agricultural sensor arrays. Reproduced with permission.^[^ ^41^ ^]^ Copyright 2022, Elsevier. b) Illustration of moisture sensor structure, composed of natural and synthetic biodegradable polymers, designed for soil moisture sensing applications. Reproduced with permission.^[^ ^30^ ^]^ Copyright 2021, American Chemical Society. c) Illustration of an array of partially biodegradable temperature sensors composed of PVA and ionic gel, designed to monitor ambient temperature. Reproduced with permission.^[^ ^71^ ^]^ Copyright 2020, American Chemical Society. d) Overview of partially biodegradable soil moisture sensor, including Sn conductive lines on nano‐paper substrates. Reproduced with permission.^[^ ^74^ ^]^ Copyright 2023, Wiley. e) Illustration of partially biodegradable flexible device featuring a chitosan composite film and an array of Fe/Mg electrodes for air humidity sensing applications. Reproduced with permission.^[^ ^77^ ^]^ Copyright 2020, The American Association for the Advancement of Science. f) Resistive temperature sensor, designed for aquatic environment applications, consists of three main components: the sensing element composed of degradable Mg electrodes, with Si_3_N_4_ and SiO_2_ as insulators. Reproduced with permission.^[^ ^42^ ^]^ Copyright 2017, Wiley.

**Figure** **2** illustrates degradable materials typically used in transient sensors for environmental applications. Figure 2a shows the powering part of an agricultural sensor array driven by wind energy, utilizing the triboelectric nanogeneration properties of corn bran material. While corn bran is a biodegradable natural polymer, the system is only partially degradable due to the presence of non‐biodegradable components, including polytetrafluoroethylene, epoxy glass, and copper.^[^ ^41^ ^]^ Corn bran, known for its wear‐resistance, moisture‐proof properties, and pollution‐free nature, degrades enzymatically with cellulase in soil.^[^ ^68^ ^]^ Methylcellulose is added as a thickener to improve the moldability and triboelectric performance of the corn husk powder film.

A partially biodegradable soil moisture sensor, shown in Figure 2b, incorporates soy and beeswax as degradable hydrophobic encapsulants alongside degradable zinc (Zn) electrodes and rapidly degradable functional components based on natural and synthetic polymers such as wood and Poly(3‐hydroxybutyrate‐co‐3‐hydroxyvalerate) (PHBV), a microbial‐driven biodegradable polymer.^[^ ^30^ ^]^ This material combination allows the sensor to function reliably in soil for a controlled period before rapid decomposition occurs following the degradation of the encapsulant. While wood decomposes only in outdoor environments through wood‐rotting fungi, PHBV is biodegradable in soil,^[^ ^69^ ^]^ making these materials ideal for environmentally sustainable applications. The thickness of the wax encapsulant determines the device's lifetime. However, increasing the encapsulant thickness to extend its lifetime significantly reduces sensitivity.^[^ ^30^ ^]^ To overcome this limitation, ultra‐thin encapsulation layers such as sputtered silicon nitride/oxide (Si_3_N_4_/SiO_2_) are proposed. These materials break down much more slowly than wax (Si_3_N_4_∼10–100 years; SiO_2_ typically hundreds of years depending on conditions), which decomposes in soil within 15–30 days. In contrast, when applied as ultra‐thin sputtered films, on the order of tens of nanometers, their degradation rates increase considerably, mainly because of the smaller material volume available for dissolution. At this thickness, Si_3_N_4_ degrades within 6–12 months, and SiO_2_ over several years. Compared to wax, these sputtered layers offer a more controlled and prolonged degradation timeline, enabling device lifetimes that are up to 10 times longer. However, their applicability may be limited to cleanroom‐fabricated devices, as sputtering and patterning processes are complex, deposition temperature can restrict substrate selection, and achieving uniform coverage over topographies remains challenging.^[^ ^64^, ^70^ ^]^

Figure 2c shows a water‐soluble ionic gel‐based environmental temperature sensor with non‐biodegradable copper electrodes, developed by Yamada et al.^[^ ^71^ ^]^ The ionic gel consists of hydrolysable polymer networks, including poly(vinyl alcohol) (PVA) as the substrate, along with poly(ethylene oxide) (PEO) and poly(ethylene glycol) (PEG), which degrade upon exposure to water (such as rainfall in the environment).^[^ ^71^ ^]^ Its performance relies on the temperature‐dependent viscosity of the ionic gel as temperature rises, viscosity decreases, enhancing ion mobility and conductivity. Between 30 and 80 °C, the sensor shows a 12‐fold conductivity and a 4.8‐fold capacitance increase.^[^ ^72^ ^]^

These results demonstrate the superior sensitivity of the ionic gel‐based sensor for high‐precision applications while highlighting the need for flexible, environment‐specific encapsulations such as biodegradable, hydrophobic, and ultra‐thin Si‐gelatine hydrogel or Ecoflex to mitigate environmental effects like moisture and wind without compromising temperature sensitivity.^[^ ^42^, ^71^, ^73^ ^]^

A partially degradable wireless soil moisture sensor is shown in Figure 2d. The sensor is constructed with patterned tin (Sn) lines functioning as a non‐biodegradable antenna, which is connected to a carbon‐based heater on a substrate made of wood‐derived cellulose nanofibers, encapsulated with wax.^[^ ^74^ ^]^ Both the substrate and the wax are degradable; the cellulose nanofibers degrade in the presence of cellulase enzymes in soil environments, while the wax degrades with lipase enzymes under similar conditions.^[^ ^75^ ^]^ Another limitation arises from potential cross‐sensitivity issues, where temperature changes could be misinterpreted as variations in soil moisture. To address this, sensor design modifications can include materials with selective moisture‐dependent properties that minimize sensitivity to temperature fluctuations.^[^ ^76^ ^]^

A partially biodegradable air humidity sensor is shown in Figure 2e. The sensor comprises degradable iron/magnesium (Fe/Mg) electrodes deposited on a composite of chitosan and lignin, which serves as the humidity‐sensitive layer supported by a flexible substrate cast from biomass. The active composite exhibits higher water absorption, forming a 3D network of proton‐conducting hydrogen bonds.^[^ ^77^ ^]^ Protons move along these chains via the Grotthuss‐type mechanism, enabling efficient proton transport. Notably, the proton current in the biodegradable composite film at 75% relative humidity (RH) is three times higher than at 65% RH, highlighting its strong sensitivity to higher humidity levels. However, the sensitivity for RH below 60% is very low due to limited water absorption, which restricts the formation of a continuous hydrogen‐bond network necessary for proton conduction. Monitoring lower humidity levels, particularly in the 20–50% RH range, is critical for effective environmental and agricultural management. In soil and crop management, monitoring air humidity, especially when it drops below 30–40% RH, is essential for detecting drought stress, as these conditions accelerate plant transpiration and soil moisture loss, threatening crop yield.^[^ ^78^ ^]^ To ensure accurate and responsive measurements in such low‐humidity environments, sensors with enhanced proton‐conducting mechanisms or alternative active materials are needed.

In Figure 2f, a partially biodegradable temperature sensor is fabricated using a degradable Mg resistive pattern encapsulated within Si_3_N_4_ insulators. The substrate and encapsulation layers are based on Ecoflex, a compostable flexible polymer certified by BASF that is known for its high water and tear resistance.^[^ ^42^ ^]^ This material is derived from corn starch, potato, and polylactic acid (PLA), and is one of the few biodegradable plastics that complies with both European Directive 2002/72/EC and American food legislation (FCN 907) for food contact applications. Ecoflex's high water filtration resistance makes it particularly suitable for temperature sensing in oceanic environments. Precise monitoring of thermal gradients is essential for studying marine ecosystems, tracking ocean currents, and understanding climate‐driven phenomena like thermal stratification or upwelling.^[^ ^79^ ^]^ The material's durability and resistance to water penetration ensure consistent performance under prolonged submersion, making it an excellent choice for such applications. Similarly, the use of amphiphobic poly(butanedithiol) (PBDT) encapsulation, as demonstrated by Choi et al. for biodegradable temperature and pressure sensors, enhances device lifetime under moist or aqueous conditions while maintaining full biodegradability.^[^ ^80^ ^]^

## 4 Degradation Mechanisms of Biodegradable Materials in Various Environments

Biodegradable materials decompose through several key mechanisms, influenced by both environmental factors and material properties. Among the most significant are fungal and bacterial degradation, where microorganisms enzymatically break down polymer structures, oxidative degradation, in which processes like ultraviolet (UV) exposure initiate chemical changes; and hydrolytic degradation, where water interacts with susceptible bonds, leading to material breakdown.^[^ ^32^, ^102^, ^103^ ^]^ The following sections examine the principles and processes underlying each of these degradation pathways in detail.

### 4.1 Fungal and Bacterial Degradations

**Figure 3:**
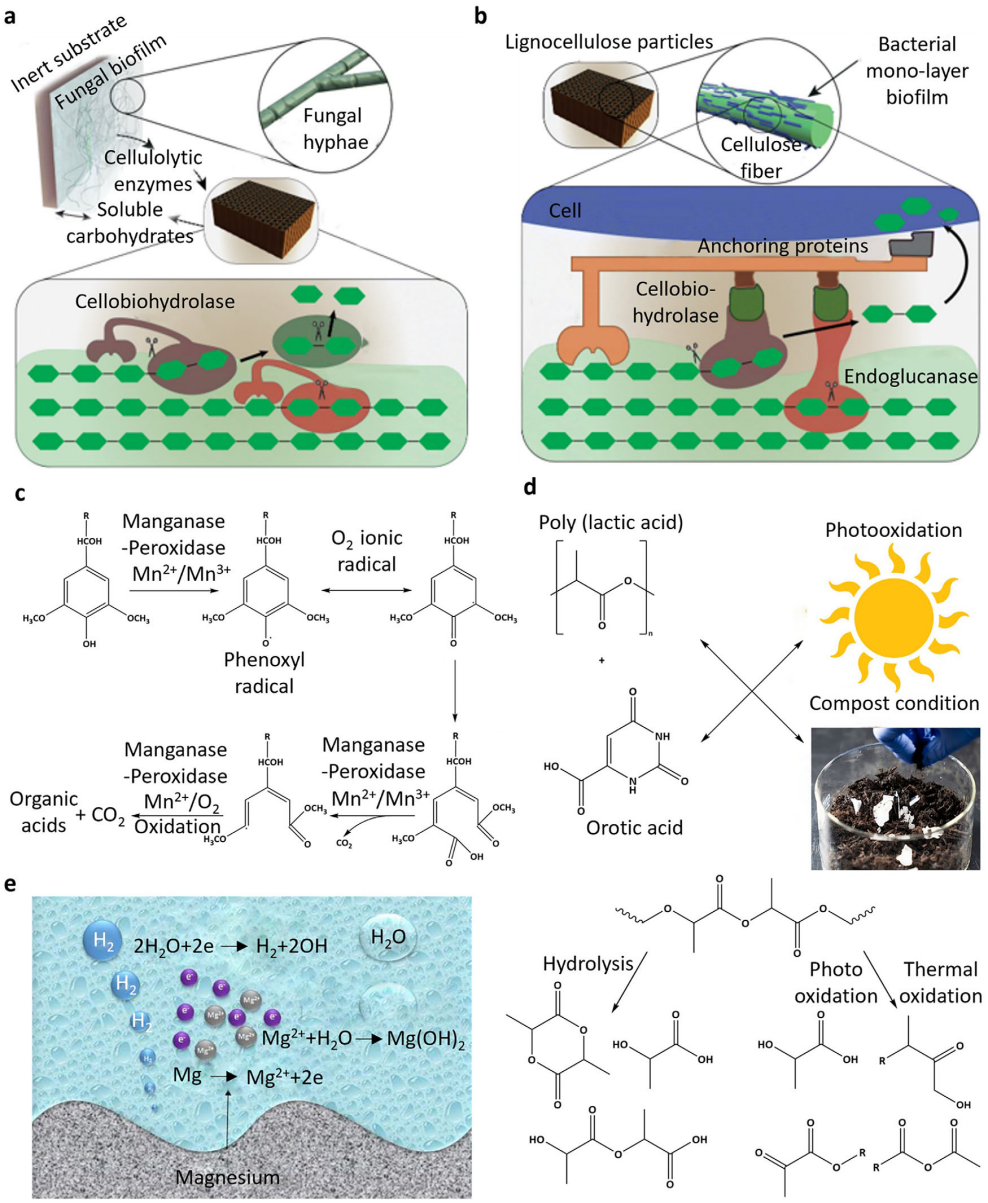
Illustration of different degradation mechanisms of biodegradable material in the environment: a) Cellulose degradation in soil facilitated by cellulolytic enzymes from fungi. Reproduced with permission.^[^ ^102^ ^]^ Copyright 2020, Springer. b) Schematic illustration of bacterial degradation of cellulose film in soil environment. Reproduced with permission.^[^ ^102^ ^]^ Copyright 2020, Springer. c) MnP enzyme‐driven breakdown of lignin in soil, leading to the release of carbon dioxide (CO_2_) during wood degradation. Reproduced with permission.^[^ ^103^ ^]^ Copyright 2002, Elsevier. d) Illustration of the photodegradation, hydrolysis, and thermal degradation of PLA in an air environment. Reproduced with permission.^[^ ^119^ ^]^ Copyright 2019, Multidisciplinary Digital Publishing Institute. e) Schematic of Mg hydrolytic degradation process in an aqueous environment. Reproduced with permission.^[^ ^120^ ^]^ Copyright 2023, Springer.

Cellulose and lignin, the primary structural components of wood, are commonly used in biodegradable sensors due to their abundance, natural origin, and readiness to break down in environmental conditions. Among these, cellulose is more easily degradable and primarily broken down by fungi and bacteria in the environment, with this process being most prominent in soil ecosystems. In contrast, cellulose degradation is less significant in air or ocean environments due to less favorable conditions for these microorganisms.^[^ ^104^, ^105^ ^]^ Fungal hyphae can be grown in submerged environments or as biofilms on inert surfaces, with biofilms reaching several millimeters in thickness (**Figure** **3**a). Cellulolytic enzymes, including endoglucanases, cellobiohydrolases, and β‐glucosidases, are produced and secreted by fungi.^[^ ^102^, ^106^ ^]^ Cellulose is hydrolyzed into glucose monomers by these enzymes, which then diffuse into the biofilm and serve as a carbon source for the fungi.^[^ ^102^ ^]^

Cellulolytic bacteria produce cellulosomes, which are enzyme complexes composed of various catalytic subunits connected through dockerin and cohesion domains to a scaffolding. These bacteria form a monolayer biofilm on lignocellulose particles or cellulose fibers to maintain close contact with the insoluble substrate, enhancing the degradation process (see Figure 3b).^[^ ^102^ ^]^ This phenomenon is more dominant in soils rich in organic matter, such as forest soils, agricultural soils, and peat soils. Forest soils, with their continuous deposition of leaf litter and plant material, and agricultural soils, enriched by crop residues, provide abundant cellulose for bacterial activity.^[^ ^107^ ^]^ Similarly, peat soils in wetlands provide organic‐rich conditions that support cellulolytic bacteria. In contrast, sandy or rocky soils, which have low organic content, show reduced cellulolytic activity due to the limited availability of cellulose substrates.

To degrade a biodegradable polymer like cellulose, the soil must provide optimal environmental conditions that support the activity of specific fungi and bacteria, the primary decomposers. Key microorganisms involved include fungi like Trichoderma reesei, Aspergillus niger, and Penicillium chrysogenum, and bacteria such as Cellulomonas spp., Streptomyces spp., and Bacillus subtilis.^[^ ^108^, ^109^ ^]^ These microorganisms thrive in soils between 25 and 35 °C, as microbial activity typically declines outside this range. Soil moisture content should be maintained at ≈50–70% of field capacity to ensure water availability for microbial processes without creating anaerobic conditions that inhibit aerobic decomposers.^[^ ^110^ ^]^ A neutral to slightly acidic pH is ideal for supporting microbial enzymatic activity. Aerobic conditions are critical, which can be achieved by maintaining a well‐drained soil structure with a 40–60% porosity.^[^ ^111^ ^]^ The carbon‐to‐nitrogen (C:N) ratio of the soil should range between 20:1 and 30:1, providing adequate nutrients for microbial growth and enzyme production. These conditions optimize the breakdown of cellulose into simpler compounds.^[^ ^112^ ^]^ While cellulose is readily broken down by fungal and bacterial enzymes, lignin presents a greater challenge due to its complex structure and resistance to microbial attack, necessitating oxidative degradation pathways facilitated by enzymes such as manganese peroxidase (MnP).^[^ ^113^ ^]^

As shown in Figure 3c, MnP—produced by basidiomycetes—is critical for breaking down lignin by oxidizing manganese(II) ions (Mn^2+^) and depolymerizing both natural and synthetic lignin.^[^ ^56^, ^114^ ^]^ Laccase (benzenediol–oxygen oxidoreductases) is another chemical found in plants, fungi, bacteria, and insects and plays a crucial role in oxidation reactions.^[^ ^103^ ^]^ The free radicals formed during these reactions, catalyzed by laccase, serve as substrates for the enzyme. Laccase facilitates the generation of phenoxyl radicals, which undergo various non‐specific reactions, including the formation of ketones, alkyl‐aryl cleavage, and demethoxylation.^[^ ^115^ ^]^ While this subsection focuses on the degradation of cellulose and lignin as representative natural polymers, similar microbial degradation mechanisms are also relevant for other biodegradable materials such as gelatin, starch‐based polymers, and casein.^[^ ^99^, ^116^, ^117^ ^]^ Moreover, fungal and bacterial activity extends beyond polymeric substrates, with applications in biomining and bioleaching processes involving environmentally degradable metals such as Mg, Fe, and Zn.^[^ ^118^ ^]^ As many biodegradable environmental sensors incorporate cellulosic or wood‐based materials, understanding their microbial degradation provides valuable insights into material selection and long‐term environmental impact.

### 4.2 Oxidative Degradation

Oxidative degradation, including photo‐oxidation and thermo‐oxidation, involves the breakdown of materials due to oxidation reactions. Photo‐oxidation, such as UV‐induced degradation, occurs when photon energy generates free radicals. This type of degradation is more prevalent in environments with high sunlight exposure, such as open agricultural fields, coastal areas, and arid regions.^[^ ^121^ ^]^ In agricultural fields, plastic mulches and films used for crop protection are particularly vulnerable due to prolonged exposure to sunlight. In coastal and ocean environments, floating plastics experience intense UV radiation, leading to photodegradation at the water's surface.^[^ ^122^ ^]^ Forests and shaded soils generally exhibit lower rates of photo‐oxidation because canopy cover or soil burial reduces direct UV exposure. Degradation in the air occurs when materials like aerosols or microplastics are suspended and exposed to sunlight for extended periods.^[^ ^112^ ^]^

Photodegradation reactions are influenced by free radical formation, as observed in the photodegradation of PLA. Incorporating catalysts like orotic acid accelerates this process, reducing the molecular weight of PLA and shortening its degradation phase (Figure 3d).^[^ ^119^ ^]^ Thermo‐oxidation is a major degradation pathway in biodegradable materials, especially in oxygen‐rich environments. Elevated temperatures induce radical formation within polymers, enhancing degradation reactions.

Multiple degradation mechanisms often operate simultaneously.^[^ ^32^, ^123^, ^124^ ^]^ Photo/thermal oxidation of biodegradable polymers, such as PLA, requires specific environmental conditions involving adequate light exposure, heat, oxygen, and moisture levels. For photodegradation, UV light intensity is a critical factor; in agricultural environments, UV radiation can range from 0.15 to 0.45 W m^−2^, depending on latitude and season.^[^ ^125^ ^]^ Thermal oxidation, on the other hand, relies on elevated temperatures that accelerate chemical breakdown. In agricultural fields with high solar exposure, surface temperatures may reach 50–70 °C, supporting oxidative processes.

In dryland environments, with temperatures often exceeding 40 °C and limited moisture (RH below 30%), thermal oxidation may dominate due to reduced microbial activity.^[^ ^126^ ^]^ Conversely, forest environments with moderate temperatures (20–35 °C) and higher humidity levels (50–80%) may slow thermal oxidation but enhance biodegradation through combined microbial activity and oxidative processes.^[^ ^127^ ^]^ The presence of oxygen is pivotal across all scenarios, whereas the oxygen diffusion rates can vary depending on polymer thickness and exposure.

Graphene‐based materials such as graphene, graphene oxide (GO), reduced graphene oxide (rGO), and laser‐induced graphene (LIG) have been reported to exhibit degradability under certain environmental conditions. Their degradation occurs through oxidative or microbial processes, particularly in the case of GO and LIG, which possess higher chemical reactivity and porous structures that facilitate breakdown.^[^ ^94^ ^]^ In contrast, carbon black can undergo partial disintegration under environmental conditions; however, the remaining carbon residues are chemically stable, inert, and generally regarded as environmentally compatible due to their low toxicity.^[^ ^94^, ^95^ ^]^

### 4.3 Hydrolytic Degradation

Hydrolysis‐driven degradation is a major pathway for both organic and inorganic materials, particularly in aqueous environments. Among metallic materials, Mg, Zn, Fe, Molybdenum (Mo), and Tungsten (W) are of particular interest for environmentally degradable sensors because their corrosion behavior facilitates degradation under moist conditions, forming corresponding hydroxides and oxides such as Mg(OH)_2_, Zn(OH)_2_, Fe(OH)_2_, Fe(OH)_3,_ MoO_4_^2−^, and WO_4_^−2^.^[^ ^128^ ^]^ The hydrolysis reaction for Mg, shown in Figure 3e, exemplifies this process, where magnesium reacts with water to form magnesium hydroxide and hydrogen gas. These processes collectively demonstrate the role of hydrolysis in the degradation of metallic materials under environmental exposure.^[^ ^120^ ^]^

Metals degrade through electrochemical corrosion rather than microbial activity; thus, the term “biodegradable metal,” commonly used in biomedical contexts, is not directly applicable to soil or aquatic environments.^[^ ^64^, ^65^, ^66^ ^]^ The degradation rate (expressed as metal thickness loss in mm year^−1^) and the environmental tolerance to the released ions both determine ecological safety.

Mg degrades the fastest among structural metals, typically 0.3–1.0 mm year^−1^ in neutral aqueous or chloride‐rich environments, and may exceed 2 mm year^−1^ in saline conditions.^[^ ^129^ ^]^ Corrosion proceeds via anodic Mg dissolution and water reduction, generating Mg(OH)_2_ and H_2_ gas with an initial burst release phase before partial passivation. Mg^2+^ is environmentally benign: the WHO drinking‐water limit is 50 mg L^−1^, and Mg‐rich soils tolerate several thousand mg kg^−1^ without toxicity.^[^ ^130^ ^]^

Zn corrodes moderately at 0.02–0.2 mm year^−1^, producing Zn(OH)_2_ and zinc oxide (ZnO) as corrosion products. Excess Zn^2+^ can inhibit microbial and plant enzymes.^[^ ^131^ ^]^ According to EU Regulation 2019/1009, the maximum allowable Zn in soil amendments is 300 mg kg^−1^, while aquatic toxicity generally occurs above 30–50 µg L^−1^.^[^ ^132^ ^]^

Fe degrades slowly, usually < 0.05 mm year^−1^, forming insoluble Fe(OH)_2_/Fe(OH)_3_ that limits ionic mobility.^[^ ^133^ ^]^ Soils naturally contain Fe in the 1–5% range (10 000–50 000 mg kg^−1^), and freshwater limits are 2–5 mg L^−1^. Thus, Fe is one of the least hazardous degradable metals environmentally.^[^ ^130^ ^]^

Mo and W exhibit very high corrosion resistance, typically < 0.001 mm year^−1^ in neutral media.^[^ ^134^ ^]^ For Mo, recent soil‐quality recommendations suggest a threshold of 3–200 mg kg^−1^, and the WHO water guideline is 70 µg L^−1^. W remains mostly insoluble in neutral soils but can form soluble WO_4_^2−^ in acidic or oxidizing conditions.^[^ ^130^ ^]^

Copper (Cu), aluminum (Al), and silver (Ag) are comparatively stable, with corrosion rates < 0.01 mm year^−1^ under neutral conditions but faster dissolution in acidic or saline environments. For Cu, EU Regulation 2019/1009 limits concentrations in soil improvers to 200 mg kg^−1^, and aquatic toxicity occurs above 20–50 µg L^−1^.^[^ ^132^ ^]^ Al becomes soluble and toxic below pH 5, with WHO water guidelines of 200–300 µg L^−1^, while Ag shows the highest toxicity, with typical ecological thresholds below 1 µg L^−1^ in water.^[^ ^130^ ^]^

Overall, while Mg, Zn, and Fe are described as “biodegradable” in biomedical contexts, their degradation in natural environments is governed purely by electrochemical corrosion. The environmental compatibility of these metals, therefore, depends on both their corrosion kinetics and the bioavailability of their degradation products, which must remain below ecotoxic limits for each intended application.

In contrast to metallic systems that corrode electrochemically, polymeric materials such as PLA and polyhydroxybutyrate‐co‐valerate (PHB‐PHV) degrade through hydrolytic cleavage of ester bonds, a process strongly influenced by environmental temperature, humidity, and microbial activity. Unlike Mg alloys, these polymers typically degrade at significantly slower rates due to their chemical structures, with degradation highly dependent on specific environmental conditions. PLA degrades primarily through hydrolysis, where water molecules break its ester bonds. While this process occurs efficiently in the human body—often within 6–12 months due to the warm, moist, and enzymatically active environment—it can take 2–5 years in nature due to limited moisture (below 20–30%), low temperatures (10–25 °C), and insufficient microbial activity.^[^ ^19^, ^32^ ^]^ Microbes involved in PLA degradation include specific bacterial species such as Pseudomonas and Bacillus, as well as fungi like Aspergillus.^[^ ^135^ ^]^ Due to its slow degradation under natural conditions, such as soil and freshwater environments, PLA typically requires 2–4 years to exhibit noticeable mass loss because of low moisture content, mild temperatures, and limited microbial activity. Effective hydrolysis of PLA generally requires elevated temperatures (above 50 °C) and microbial assistance from species such as Thermomonospora and Streptomyces; under these conditions, complete degradation can occur within a few months.^[^ ^136^ ^]^ Industrial composting at temperatures of 55–70 °C, RH of 50–60%, and with targeted microbial consortia further accelerates degradation to 30–90 days.^[^ ^137^ ^]^

PHB‐PHV biodegrades more effectively in soils and marine environments under optimal conditions, at 30–50 °C, RH above 40%, and microbial activity from species like Cupriavidus necator. The degradation rate is significantly reduced in nutrient‐poor environments, taking 1–3 years.^[^ ^138^ ^]^

Research has focused on polymer blending, catalyst incorporation, and structural modifications to enhance degradation rates under ambient conditions, while also expanding composting infrastructure for large‐scale applications.^[^ ^139^ ^]^ However, controlling the degradation of biodegradable sensors remains crucial for their reliability and sustainability. Factors such as moisture, temperature, and UV exposure significantly influence degradation rates, yet the absence of standardized testing hampers performance comparisons. While hydrolysis dominates the degradation of many polyesters such as PLA and PHB‐PHV, other mechanisms—such as enzymatic and microbial decomposition—are equally relevant for materials like cellulose, chitosan, and lignin‐based composites. The dominant degradation pathway depends strongly on both material composition and environmental conditions: hydrolytic mechanisms prevail in aqueous environments, whereas microbial or fungal activity dominates in soils and compost. Combining materials with complementary degradation mechanisms can therefore enable sensors optimized for specific end‐of‐life scenarios. **Table** **3** summarizes the biodegradable materials discussed, highlighting their primary degradation mechanisms, relevant environments, and key influencing factors.

**Table 3:** (Bio)degradable materials and their primary degradation mechanisms in various environments.

Material	Degradation mechanisms	Typical environment	Key factors	Refs.
Cellulose	Enzymatic hydrolysis by fungi and bacteria (endoglucanases, cellobiohydrolases, β‐glucosidases)	Soil, compost	Moisture (50–70%), temp 25–35 °C, aerobic, C:N 20–30:1	[102, 110, 112]
Lignin	Oxidative degradation (MnP, and radical oxidation)	Soil, forest litter	Ligninolytic fungi (basidiomycetes), oxygen	[103, 113]
PLA	Hydrolysis of ester bonds (slow in soil, faster in compost)	Soil, compost	Temp 55–70 °C, RH 50–60%, microbial consortia UV intensity (0.15–0.45 W/m^2^), surface temperatures 40–70 °C, oxygen	[119, 125, 126]
PHB‐PHV	Hydrolysis + microbial degradation (Cupriavidus necator, fungi)	Soil, marine, compost	Temp 30–50 °C, RH >40%	[136, 138]
GO, rGO, and LIG	Oxidative or microbial oxidation	Aqueous, aerobic	Breakdown promoted by high oxygen content and porosity; inert carbon residues remain	[94, 95]
Mg	Hydrolysis/corrosion (anodic oxidation, H_2_ release)	Aqueous, soil	pH, oxygen, moisture	[120]
Zn	Hydrolytic/corrosion	Soil, Aqueous	Controlled by pH and chloride; EU* soil limit = 300 mg kg^−1^; aquatic toxicity > 30–50 µg L^−1^	[131, 132]
Fe	Hydrolytic/corrosion	Soil, Aqueous	Stable under neutral pH; soil background = 10 000–50 000 mg kg^−1^; WHO* water = 2–5 mg L^−1^	[130, 133]
Mo / W	Hydrolytic/corrosion	Soil, Aqueous	Stable in neutral media; soluble anions MoO_4_^2−^ / WO_4_^2−^ form in acidic or oxidizing conditions; Mo soil limit = 3–200 mg kg^−1^; WHO water = 70 µg L^−1^	[130, 134]
Cu	Hydrolytic/corrosion	Soil, Aqueous (acidic/saline)	Soluble below pH 6; EU soil limit = 200 mg kg^−1^; aquatic toxicity > 20–50 µg L^−1^	[130, 132]
Al	Hydrolytic/corrosion (acidic dissolution)	Acidic soil, Aqueous	Soluble and toxic at pH < 5; WHO water = 200–300 µg L^−1^	[130]
Ag	Hydrolytic/corrosion	Aqueous	Toxic above 1 µg L^−1^ (WHO aquatic threshold); Limited solubility at neutral pH	[130, 132]

EU: European Union; WHO: World Health Organization.

## 5 Biodegradable Devices for Ecosystem and Environmental Monitoring Applications

Biodegradable sensors provide a sustainable and practical approach to environmental monitoring, particularly in agriculture, pollution control, and climate research.^[^ ^15^ ^]^ They are used to measure parameters such as soil moisture, pH, nutrient levels, temperature, light intensity, and chemical pollutants, generating valuable data for resource management and ecosystem assessment.^[^ ^21^, ^45^, ^140^, ^141^ ^]^ Their degradability is particularly beneficial for temporary or hard‐to‐retrieve deployments in crop fields, water bodies, and remote ecosystems, where collecting conventional devices is impractical or environmentally intrusive.^[^ ^15^ ^]^ Compared with non‐degradable sensors, biodegradable devices can reduce electronic waste and lower the environmental footprint of short‐term or single‐use monitoring campaigns. LCA studies support their potential environmental advantages in specific contexts where avoiding retrieval or minimizing residue is more sustainable than reuse or recycling.^[^ ^9^, ^14^, ^142^ ^]^ Although long‐term benefits depend on the application and disposal conditions, biodegradable sensors complement reusable systems by enabling low‐impact, localized monitoring in sensitive environments. Depending on their design, these sensors can operate as chipless passive devices that rely on resonance and backscattering mechanisms for wireless signal transmission, or as electronics‐integrated systems incorporating minimal circuitry for active sensing and data communication.^[^ ^32^ ^]^ The following sections explore their specific applications and the impact of their degradability on functionality and long‐term environmental benefits.

### 5.1 Moisture and Humidity Sensors

RH sensors measure air moisture relative to saturation at a given temperature, while soil moisture sensors quantify water content below the surface, both supporting efficient irrigation and optimal root‐zone conditions.^[^ ^143^, ^144^ ^]^ Monitoring soil and atmospheric moisture is crucial for regulating irrigation, managing greenhouse climates, and mitigating drought stress, thereby enhancing crop yield and conserving water resources.^[^ ^143^ ^]^ Collectively, they enable precision farming approaches such as drip irrigation, controlled‐environment agriculture, and early drought detection, thereby improving resource efficiency and supporting sustainable water management.^[^ ^74^, ^145^ ^]^

**Figure 4:**
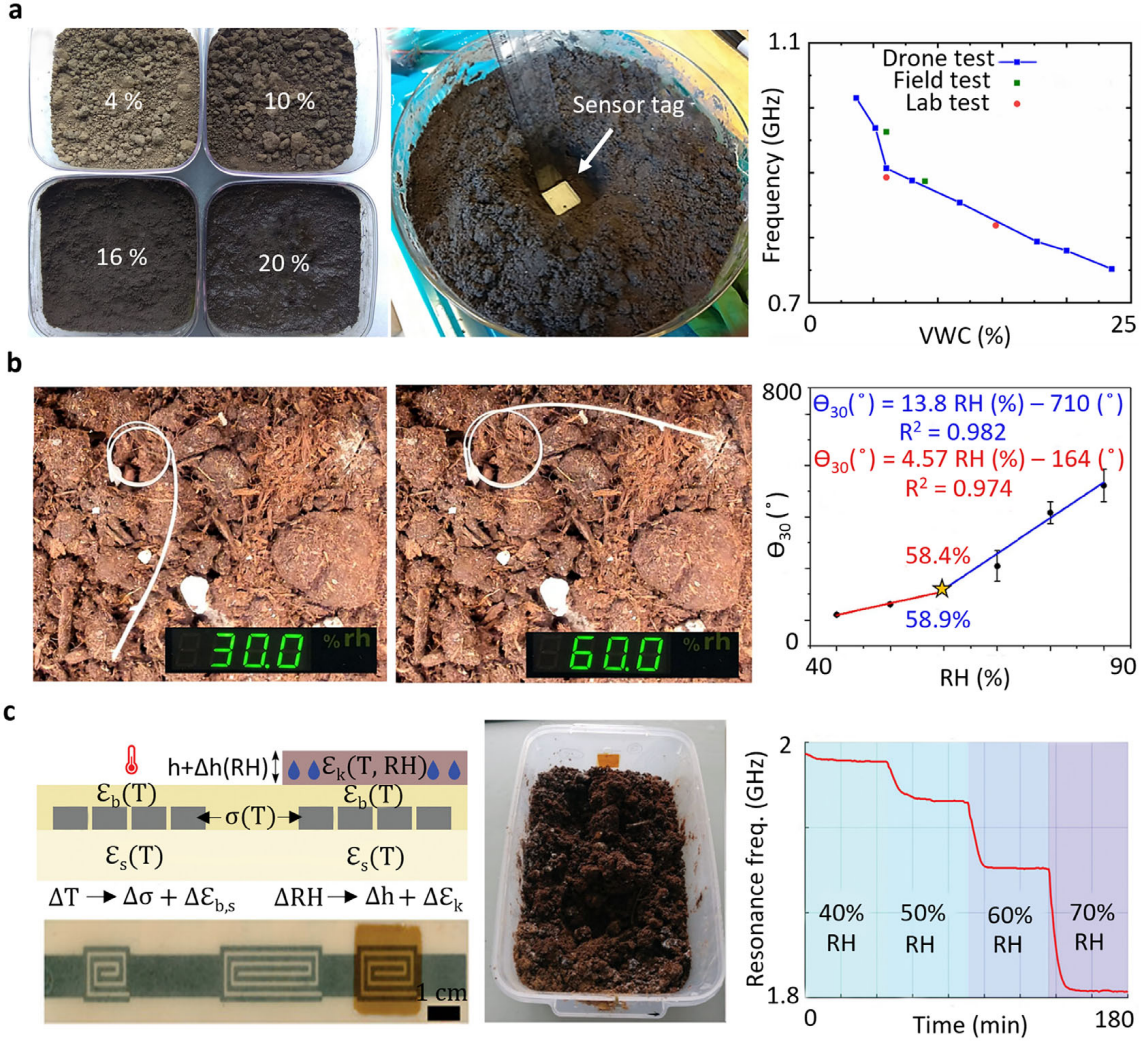
(Bio)degradable moisture and RH sensors for environmental monitoring applications: a) Partially biodegradable, wireless, and chipless sensor fabricated with laser cutting for agricultural applications to monitor soil's volumetric water content. Reproduced with permission.^[^ ^32^ ^]^ Copyright 2022, Springer Nature. b) Fully passive, chipless hygroscopic RH sensor in air, utilizing 4D‐printed natural Pelargonium appendiculatum seeds and designed for ecological monitoring. Reproduced with permission.^[^ ^22^ ^]^ Copyright 2023, Elsevier. c) Partially biodegradable, printed RH chipless sensor, featuring a microstrip line resonator with screen printed degradable Zn on a paper substrate encapsulated in beeswax, is designed for sustainable monitoring of soil and ecological conditions.^[^ ^90^ ^]^ Copyright 2024, Wiley.

In this context, (bio)degradable moisture and RH sensors are increasingly necessary for short‐term and seasonal agricultural deployment, where sensor networks—typically 50–200 nodes per hectare—are installed for 2–4‐month crop cycles. Nondegradable sensors generate high environmental footprints and are rarely retrieved after use. LCA studies show that printed biodegradable humidity tags reduce cradle‐to‐gate global warming potential by ≈39% (from 42 to 25.7 g carbon dioxide equivalent (CO_2_e) per tag) compared to standard devices, while nondegradable sensors reach ≈613 g CO_2_e per unit, over 20 times higher.^[^ ^13^, ^146^ ^]^ Their short functional lifetime aligns with seasonal monitoring needs, allowing deployment throughout cultivation periods without contributing to long‐term waste.^[^ ^147^ ^]^ However, the (bio)degradable moisture and RH sensors are still at a research and development stage, and further studies are needed to enhance their stability, reliability, and scalability for real‐world agricultural applications, as illustrated in **Figure** **4**.

The partially biodegradable, wireless, passive, and chipless sensor developed by Gopalakrishnan et al.,^[^ ^32^ ^]^ illustrated in Figure 4a, offers an innovative approach to soil moisture monitoring by utilizing PLA and degradable Zn foil to detect changes in the dielectric constant of the environment. The sensor operates effectively within a volumetric water content range of 4–23.5%, with a sensitivity of 9 MHz/%. However, this sensitivity may not adequately detect subtle moisture variations, particularly in heterogeneous or extreme soil conditions. The chipless passive tag radio‐transmitting sensor achieves 40 cm read distance by leveraging a backscattering mechanism at a frequency matching the tag's resonance, enabled by efficient energy harvesting and antenna design. This enables wireless electronics‐free monitoring of near‐surface soil environments. Biodegradation was demonstrated in the microbial‐rich soil environment. The sensor maintained stable signal characteristics for up to 20 days of soil exposure, after which gradual attenuation of the reflected resonance amplitude was observed due to Zn oxidation and degradation of the PLA substrate, marking the transition from reliable to unreliable readings. Biodegradation occurred progressively through microbial activity, with complete physical disintegration observed after ≈45 days in moist soil.

In comparison, Zaccarin et al.^[^ ^82^ ^]^ developed a capacitive soil moisture sensor using a cellulose‐based substrate infiltrated with cellulose nanofibrils (CNFs). The CNFs enhance dielectric properties, improving sensitivity and response time. The capacitor can be paired with an inductor to form an LC resonant circuit, enabling wireless interrogation via inductive coupling. This provided greater operational flexibility than Gopalakrishnan's design. Silva et al.^[^ ^148^ ^]^ introduced a metamaterial‐inspired microwave sensor with partially biodegradable components, detecting soil moisture between 0% and 20% with relative errors of only 3.6%. Dahal et al.^[^ ^149^ ^]^ fabricated sensors using PHBV, which completely degraded within 30 days in soil, emphasizing environmental compatibility. In terms of energy efficiency, Amiri et al.^[^ ^150^ ^]^ presented a radiofrequency (RF) self‐powered metamaterial‐based sensor capable of harvesting 65 and 100 µW at 5% and 25% soil moisture levels, respectively, allowing autonomous operation over extended periods.

The fully biodegradable and chipless hygroscopic sensor developed by Mariani et al.^[^ ^22^ ^]^ illustrated in Figure 4b utilizes natural Geraniaceae seeds to achieve angular displacement for real‐time RH monitoring. Its ability to rotate by 500 degrees under 90% humidity highlights its sensitivity and environmental adaptability. However, the reliance on visual recording via a drone limits operational efficiency for continuous or large‐scale monitoring, while its performance in extreme or rapidly changing humidity conditions remains untested.

The Geraniaceae seed‐based sensor, derived from the seeds of plants in the Geranium family, stands out for its biodegradability, providing a significant environmental benefit compared to non‐biodegradable sensors constructed from synthetic materials.

For instance, a gelatin‐based humidity sensor achieved fast response/recovery times of 4/6.3 seconds over a wide operating range of 15–86% RH.^[^ ^151^ ^]^ Despite its innovative design, the Geraniaceae sensor exhibits slower response times and potential challenges in reliability under fluctuating conditions. In contrast, polymer‐coated fiber‐based sensors and gelatin‐based systems illustrate greater consistency and speed.^[^ ^152^ ^]^

The partially biodegradable, chipless RH sensor shown in Figure 4c employs a microstrip resonator coated with konjac glucomannan, a natural, water‐soluble polysaccharide extracted from the Amorphophallus konjac root. Biodegradation was demonstrated in soil, where the sensor maintained stable operation during the first two weeks with consistent and reproducible frequency responses. On week 2, the zinc layer, no longer protected by the wax encapsulation, began to oxidize, reducing signal amplitude. By week 5, the paper substrate showed structural degradation, and by week 10, it had fragmented completely, marking the onset of unreliable readings. Without protection, the zinc microstrip exhibited a resistance drift of ≈60% after 12 weeks, whereas the introduction of beeswax encapsulation reduced this drift to below 2%, confirming its effectiveness in extending sensing reliability. Known for absorbing up to 200 times its weight in moisture, konjac glucomannan swells in humid conditions, altering the dielectric properties of the coating and thereby shifting the resonant frequency of the sensor. This passive and wireless mechanism enables humidity detection without electronic components. The sensitivity could be further increased by simply decreasing the thickness of the encapsulation layer. With a sensitivity range of −0.8 to −8 MHz/%RH between 30% and 70% RH, it effectively captures moderate humidity fluctuations. Degradable slow‐wave substrate‐integrated waveguide resonators detect a broader RH range of 30% to 90% with higher efficiency.^[^ ^153^ ^]^ Modifying the microstrip resonator design to include multi‐resonant structures or complementary split‐ring resonators could also improve sensitivity and expand the RH detection range.^[^ ^154^ ^]^

### 5.2 pH Sensors

In agriculture, pH sensors are critical for managing soil acidity, nutrient solubility, and fertilizer efficiency, yet conventional non‐degradable probes pose challenges due to high cost, complex retrieval, and accumulation of electronic waste after short‐term use.^[^ ^155^ ^]^ Biodegradable alternatives offer a sustainable solution for periodic soil assessment, particularly in seasonal crop rotations where sensors are deployed for limited durations. LCAs of biodegradable pH sensors show that reusing the readout unit while replacing only the degradable sensing patch can reduce total environmental impact by 66% for quarterly replacement and up to 79% over five years compared with fully disposable, non‐degradable devices.^[^ ^156^ ^]^

**Figure 5:**
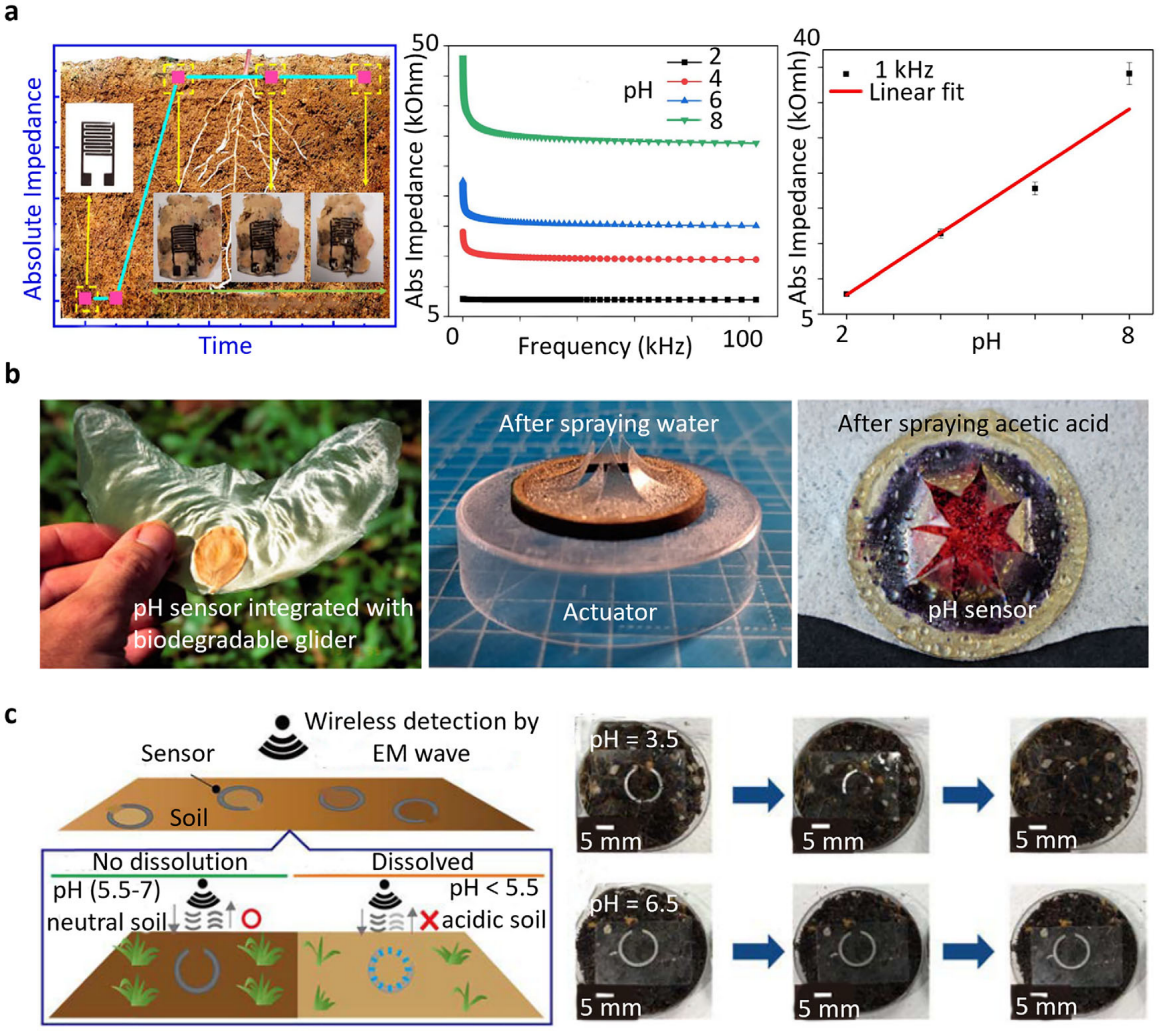
Application of (bio)degradable pH sensor for soil acidity/basicity monitoring: a) Degradable printed pH sensor monitoring soil samples with a linear impedance response (pH 2–8). Reproduced with permission.^[^ ^48^ ^]^ Copyright 2022, Institute of Electrical and Electronics Engineers (IEEE). b) Biodegradable chipless glider incorporated with a transient pH sensor covered with a water‐sensitive actuator, fabricated with plotter cutter and spray coating for monitoring rainwater acidity in air and natural ecosystems. The color change from blue to red in the exposed area of sensor after spraying the acid. Reproduced with permission.^[^ ^21^ ^]^ Copyright 2022, Frontiers. c) Conceptual illustration of a partially biodegradable and chipless soil pH sensor based on a laser‐cut ring oscillator and the related dissolution of the sensor in acid and normal soil, respectively. Reproduced with permission.^[^ ^39^ ^]^ Copyright 2021, Institute of Electrical and Electronics Engineers (IEEE).

As illustrated in **Figure** **5**a, a partially degradable pH sensor developed by Aliyana et al.^[^ ^48^ ^]^ employs a screen‐printed mixture of degradable graphene and ZnO, achieving high sensitivity of ≈5.27 kΩ per unit change in pH across the pH 2–8 range. The biodegradable paper substrate of this device decomposes completely within 45 days in compost soil, preventing solid waste accumulation after a single crop cycle. A soil burial degradation test confirmed progressive disintegration over this period, with samples washed and examined at regular intervals. After 10 days, a sharp change in electrode impedance was observed, indicating the onset of drift and unreliable measurements as the degradable electrode structure began to weaken. Integrated wireless data transmission via a nondegradable Wi‐Fi module enables real‐time soil monitoring, demonstrating the potential of degradable pH sensors to support sustainable soil management, fertigation optimization, and environmentally benign precision agriculture.

The Aliana et al.’s sensor illustrates robust performance within its designated range, but it falls short in terms of sustainability. Replacing graphene with biodegradable alternatives such as ZnO can enhance sustainability while maintaining functionality.^[^ ^157^ ^]^

As described by Bressi et al.,^[^ ^157^ ^]^ the sustainability of the graphene‐ZnO sensor can be enhanced by replacing graphene with other biodegradable alternatives, such as LIG from lignocellulosic precursors or biodegradable polymer composites.^[^ ^157^, ^158^, ^159^ ^]^ Although LIG is not inherently more degradable than pristine graphene, its porous, defect‐rich morphology and renewable, low‐energy fabrication process make it more environmentally sustainable.^[^ ^94^ ^]^ Reinforcing the stability of the active layer with protective coatings, composite materials, or encapsulation techniques further enhances its durability in complex and variable soil conditions.^[^ ^160^ ^]^

Beyond soil applications, pH sensors also play a critical role in monitoring air and natural ecosystems by assessing the impact of acid rain and environmental pollution. The pH‐reactive fully biodegradable layer integrated into a chipless glider, as illustrated by Wiesemüller et al.,^[^ ^21^ ^]^ presents a sustainable approach for monitoring rainwater acidity (see Figure 5b). Under ISO 20200‐compliant composting at 58 °C, the glider airframe degraded almost entirely within the first week, while the actuator and cellulose substrate required longer for disintegration. During this phase, readings remained stable, but as the substrate structure weakened, uneven moisture uptake caused drift and unreliable colorimetric response. The use of a litmus‐based pH‐sensitive layer provides a straightforward and cost‐effective method for visually assessing environmental pH levels. However, the reliance on visual color change for data collection reduces the system's precision, making it less suitable for applications that require continuous or remote monitoring. Biodegradable PEG hydrogel‐coated on non‐biodegradable optical fiber sensors described by Yin et al.^[^ ^161^ ^]^ achieve sensitivities of −199 pm/pH over a pH 2–6 range, with real‐time distributed measurement capabilities.

Hori et al.^[^ ^39^ ^]^ introduced a split‐ring resonator‐based partially biodegradable chip/wireless pH sensor, offering a sustainable solution for detecting acidic soil conditions, as illustrated in Figure 5c. The sensor utilizes a hydroxyapatite layer as a protective coating that dissolves in acidic environments, gradually exposing the degradable Mg ring, which acts as the sensing element. This design allows for real‐time and wireless monitoring through resonance frequency changes detected by a transmitter‐receiver antenna. Degradation verified in moist soil. The Mg sensing ring degraded within 2–3 days for films of 1–2 µm thickness, while thicker (10 µm) layers persisted up to five days; after degradation, reliable readings were no longer possible due to loss of resonance stability. However, the rapid degradation of Mg limits the sensor's operational lifespan, particularly in highly acidic soils, which can reduce its effectiveness for prolonged applications. Alternatively, optofluidic ring resonator systems represent another design approach, offering higher precision and a linear detection range of pH 6.51–8.13, making them more suitable for applications that require high accuracy and extended functionality.^[^ ^162^ ^]^

To extend the lifetime of biodegradable pH sensors, materials like patterned octacalcium phosphate coatings can be used due to their slow, controllable degradation and pH‐buffering ability. This helps maintain sensor stability in moist, acidic environments while ensuring environmental compatibility.^[^ ^163^ ^]^ In chipless resonator designs, data are transmitted via electromagnetic backscattering, and the most significant improvement can be achieved through metasurface engineering. By tailoring the resonator layout and periodic surface features, metasurfaces can concentrate electromagnetic fields, increase quality factors (Q), and boost backscattered signal strength, resulting in higher readout sensitivity and extended interrogation range for reliable wireless detection.^[^ ^164^ ^]^

The pH sensors discussed employ diverse sensing mechanisms, each with distinct advantages and limitations. Resistive sensors, such as the ZnO‐based type, detect pH through measurable changes in resistance, offering simplicity but limited resolution. Nanowire field‐effect transistor‐based (NWFET) sensors (e.g., ZnO NWFETs) operate by modulating surface potential in response to pH variations, enabling higher sensitivity and real‐time monitoring. Capacitive and resonant sensors, including split‐ring resonators, rely on shifts in capacitance or resonance frequency, facilitating wireless, selective detection suitable for remote sensing. Optical sensors, such as PEG‐coated fiber optics and optofluidic ring resonators, utilize pH‐induced spectral shifts for highly precise measurements, though they typically require more complex instrumentation. These varied mechanisms highlight the trade‐offs between sensitivity, sensing mechanism, material selection, and system complexity, guiding the choice of technology based on application requirements.

### 5.3 Chemical Sensors

Monitoring hazardous chemicals in liquid and vapor form is vital for safeguarding ecosystems and ensuring agricultural sustainability. Contaminants such as heavy metals, pesticides, and industrial pollutants threaten soil, water, and air quality, affecting crop health and food safety.^[^ ^165^ ^]^ Detecting pollutants such as lead and mercury in water or glyphosate in croplands prevents toxic accumulation in crops, livestock, and soil.^[^ ^166^, ^167^, ^168^ ^]^ Likewise, monitoring sulfur dioxide and nitrogen oxides mitigates air quality deterioration and associated ecological and health risks.

LCAs of screen‐printed electrochemical (EC) sensors reveal that the substrate is the dominant environmental hotspot, with paper substrates contributing ≈1.5 kg CO_2_e for the production stage (climate change impact excluding biogenic carbon), whereas high‐density polyethylene (HDPE) substrates show the lowest impacts in 13 of 19 categories.^[^ ^169^ ^]^ These findings demonstrate that using bio‐based, low‐energy, and degradable substrates can markedly reduce the life‐cycle footprint of disposable chemical sensors. In addition, noble‐metal electrodes contribute orders‐of‐magnitude higher impacts than carbon‐based inks, reinforcing the need for (bio)degradable, metal‐lean architectures.^[^ ^169^ ^]^ Such transient sensors, optimized for ammonia, nitrite, and pesticide vapor detection, are typically deployed for 4 to 10‐week monitoring period during fertilization, irrigation, and pesticide application in paddy soils, greenhouses, and open croplands.^[^ ^169^, ^170^, ^171^ ^]^ Their operational lifetime aligns with these seasonal agricultural cycles, providing an efficient and environmentally compatible alternative to conventional non‐degradable chemical sensing systems.^[^ ^172^ ^]^ The fully and partially biodegradable sensors presented in this section are integrated with non‐biodegradable readout systems for signal processing and data acquisition.

**Figure 6:**
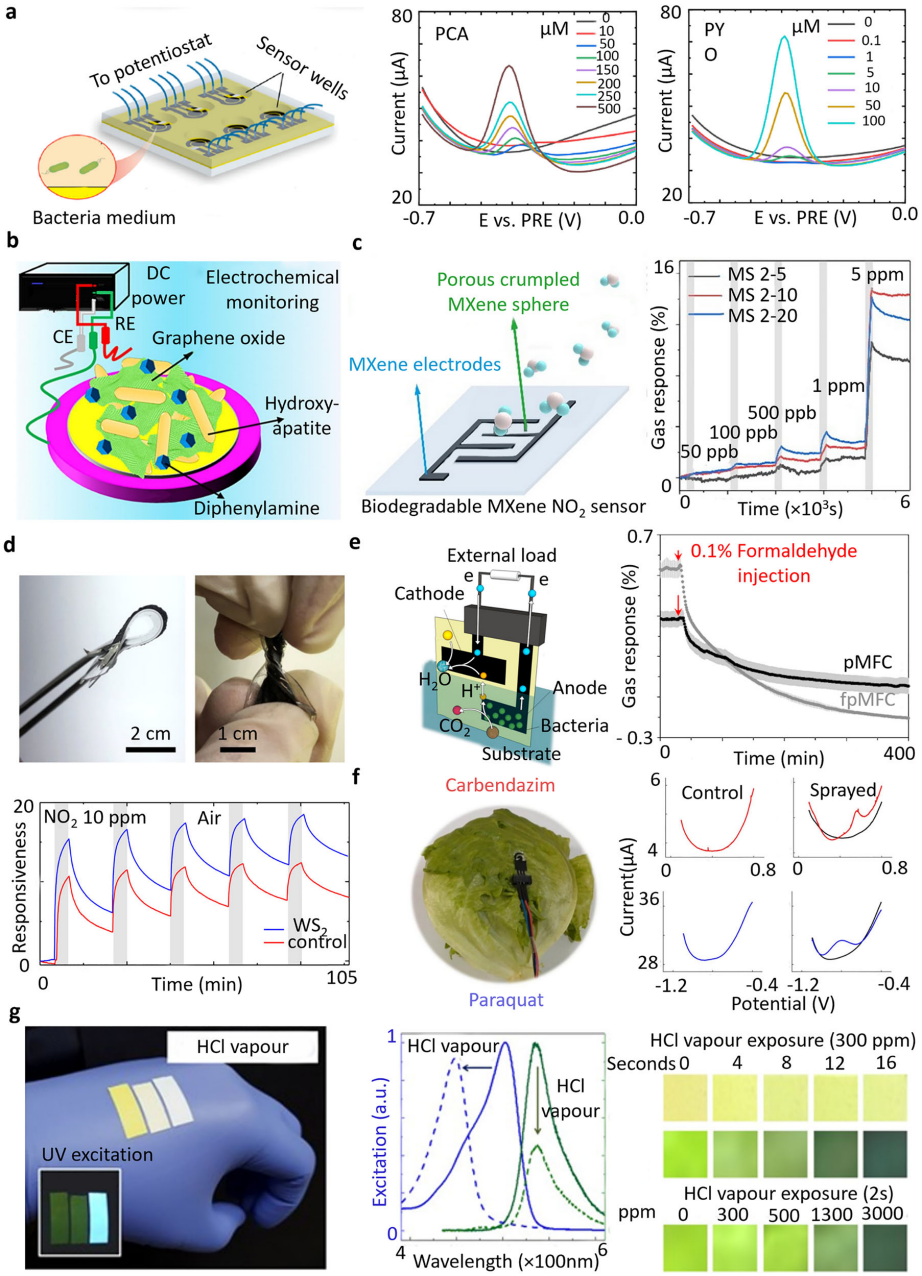
Applications of degradable sensors for various hazardous chemical detection in the environment: a) Cellulose‐based degradable EC sensor with laser‐induced graphene (LIG) electrodes for monitoring PCA and PYO as detrimental bacteria in soil. Reproduced with permission.^[^ ^50^ ^]^ Copyright 2023, Elsevier. b) Schematic of a partially biodegradable printed sensor for monitoring of DPA as a common pollutant in air and soil. Reproduced with permission.^[^ ^174^ ^]^ Copyright 2023, Elsevier. c) Degradable screen printed NO_2_ gas sensor and the related dynamic response–recovery curve in the different NO_2_ concentrations. Reproduced with permission.^[^ ^49^ ^]^ Copyright 2022, Springer. d) Flexible and degradable NO_2_ sensor based on dip‐coated TMDs electrodes. Reproduced with permission.^[^ ^184^ ^]^ Copyright 2019, American Chemical Society. e) Paper‐based screen‐printed chemical sensor for the detection of formaldehyde as a major contaminant of the soil. Reproduced with permission.^[^ ^185^ ^]^ Copyright 2018, Elsevier. f) Cellulose acetate‐based screen‐printed sensor for monitoring pesticides on the lettuce. Reproduced with permission.^[^ ^186^ ^]^ Copyright 2023, Elsevier. g) Biodegradable electrospun optical sensor based on silk nanofibers for hydrochloric acid (HCl) vapor detection. Reproduced with permission.^[^ ^141^ ^]^ Copyright 2017, Springer Nature. All sensors include integrated or external non‐degradable electronic components for readout and signal processing.

In agriculture, monitoring hazardous chemicals is crucial to prevent crop diseases and maintain soil health.^[^ ^173^ ^]^ The EC bacterial sensor, as illustrated in **Figure** **6**a, operates on a potentiometric sensing principle and utilizes degradable LIG electrodes on a cellulose substrate to detect harmful bacterial metabolites such as phenazine‐1‐carboxylic acid (PCA) and pyocyanin (PYO). A silver epoxy layer was applied to the LIG electrode to serve as a reference electrode, enabling stable potential measurements but making the device partially biodegradable. With detection limits of 500 µm for PCA and 100 µm for PYO, it offers a promising tool for early intervention to prevent plant diseases and reduce environmental damage.^[^ ^50^ ^]^

The degradable EC sensor for diphenylamine (DPA) detection, as shown in Figure 6b, incorporates a hydroxyapatite–graphene oxide composite and achieves a low limit of detection LOD of 0.009 µm.^[^ ^174^ ^]^ EC sensors based on metal‐organic frameworks (MOFs), such as those described by Xie et al.,^[^ ^175^ ^]^ achieve a limit of detection (LOD) in the range of femtomolar concentrations, significantly outperforming the PCA and PYO sensors regarding specificity and sensitivity. To address current limitations, nanopore electrode arrays described by Jia et al.^[^ ^176^ ^]^ enhance specificity for PCA in mixed bacterial environments, enabling LOD at nanomolar concentrations. Future sensor designs could incorporate hybrid materials such as molybdenum‐based composites to enhance conductivity and sensitivity while maintaining biodegradability.^[^ ^177^ ^]^ Partially biodegradable EC sensors, such as those reported by Nguyen et al., utilizing hybrid MoS_2_ nanosheets, achieve lower LOD for pollutants like DPA, ensuring higher accuracy in complex matrices.^[^ ^178^ ^]^

In natural ecosystems, monitoring harmful gases can provide valuable insights into pollution sources and air quality, contributing to a better understanding of their potential effects on biodiversity, habitat health, and overall ecological balance.^[^ ^179^ ^]^ The partially degradable nitrogen dioxide (NO_2_) sensor developed by Yang et al.^[^ ^49^ ^]^ illustrates significant potential for addressing the environmental and agricultural challenges posed by NO_2_ pollution. By utilizing porous MXene electrodes—though not biodegradable—on a PVA substrate, as shown in Figure 6c, the device effectively detects and recovers across a broad range of NO_2_ concentrations, highlighting its versatility and eco‐friendliness due to the biodegradable substrate. Upon immersion in aqueous media, visible distortion of the sensor was observed as the PVA substrate began to dissolve, indicating the onset of structural degradation. The PVA layer dissolved completely within 60 min, followed by the gradual disintegration of the porous MXene electrodes over ≈6 h. Similarly, recent studies have explored graphene derivative–based gas sensors integrated with biopolymers such as chitosan, bacterial nanocellulose, and starch for detecting gases such as NO_2_ and NH_3_ under ambient conditions.^[^ ^180^, ^181^, ^182^ ^]^ The partially degradable flexible NO_2_ gas sensor incorporating functionalized multi‐walled carbon nanotubes (MWCNTs) with nanolayered transition metal dichalcogenides (TMDs), as depicted in Figure 6d, enhances sensitivity. However, it is important to note that MWCNTs are not biodegradable and may pose long‐term environmental risks if released into ecosystems.^[^ ^183^ ^]^

Molybdenum disulfide (MoS_2_)/ZnO nanohybrid‐based sensors show response times as low as 50 ms for sub‐ppb NO_2_ levels under UV activation, providing dynamic performance superior to the non‐biodegradable MXene‐based designs.^[^ ^187^ ^]^ Future designs should focus on replacing non‐biodegradable electrodes like MWCNTs with more sustainable alternatives, such as hybrid MoO_3_‐based composites.^[^ ^188^ ^]^

Formaldehyde is a harmful environmental pollutant that poses serious health risks to humans, including respiratory issues and cancer, and negatively impacts wildlife by contaminating air and water. The degradable sensor developed by Chouler et al.^[^ ^185^ ^]^ for formaldehyde detection offers a sustainable solution to mitigate the harmful impacts of formaldehyde on environmental and agricultural systems. Using a degradable EC sensor based on screen‐printed carbon electrodes on a paper substrate, as illustrated in Figure 6e, the sensor combines simplicity with biodegradability. However, its sensitivity, indicated by a current change of 0.2 µA for 0.1% v/v formaldehyde, is insufficient for detecting lower concentrations typically found in natural environments. This limitation restricts the sensor's effectiveness for large‐scale environmental monitoring. Improving current output in EC sensors strongly depends on electrode geometry. Patterns such as interdigitated, porous/3D, or fractal‐like electrodes greatly enlarge the electroactive surface and shorten ion‐diffusion paths, thereby amplifying current response and sensitivity. Reports confirm that such optimized geometries markedly enhance the performance of biodegradable electrochemical sensors.^[^ ^189^, ^190^, ^191^ ^]^

Monitoring pesticides is crucial for sustainable agriculture and environmental health, as chemicals like carbendazim and paraquat pose significant risks.^[^ ^192^, ^193^ ^]^ The partially biodegradable, plant‐wearable EC sensor, featuring a screen‐printed carbon electrode on a cellulose acetate substrate (Figure 6f), provides a sustainable solution with detection limits of 54.9 nm for carbendazim and 19.8 nm for paraquat.^[^ ^186^ ^]^ However, its narrow detection range (0.1 to 1.0 µm) and potential selectivity limitations restrict its effectiveness in environments with varying pesticide concentrations. A degradable leaf‐wearable sensor was recently developed to monitor methanol emissions from plants under stress conditions such as water deficit, wounding, and light exposure. Fabricated by screen‐printing carbon electrodes on PVA and corn starch substrate and coating them with zinc oxide nanorods, the sensor operated at room temperature with a sensitivity of 0.1718 pF/ppm and LOD of 6.79 ppm, demonstrating strong potential for on‐plant stress monitoring in smart agriculture.^[^ ^194^ ^]^

Integrating degradable MOFs into EC sensors enhances sensitivity and selectivity due to their high surface area and porosity. A notable example is zeolitic imidazolate frameworks (ZIF‐8), a degradable MOF made from zinc ions and 2‐methylimidazole, which can degrade in mildly acidic conditions.^[^ ^195^ ^]^

Detecting volatile acid vapors, such as HCl, is crucial for protecting agricultural crops and maintaining air quality. These vapors can severely damage plant tissues, alter soil pH, and contribute to air and water pollution.^[^ ^196^ ^]^ The fully transient optical sensor developed by Min et al.^[^ ^141^ ^]^ (as shown in Figure 6g) presents a sustainable solution for monitoring these hazardous compounds. Designed with electrospun silk nanofibers doped with an organic dye, the sensor enables both visual and spectral detection, ensuring biodegradability. While practical for certain applications, the sensor's detection threshold of 300 ppm is insufficient for identifying lower concentrations that could accumulate and harm ecosystems.^[^ ^197^ ^]^

The transient silk‐based sensor's reliance on organic dyes provides a biodegradable design but raises concerns regarding the long‐term stability and durability of the nanofiber matrix in acidic conditions, where the pH is ≈4. To improve its performance, future iterations could integrate hybrid materials, such as combining silk nanofibers with robust polymers or MOFs.^[^ ^198^ ^]^

### 5.4 Temperature Sensors

Temperature monitoring is essential in agricultural, environmental, and ecological contexts.^[^ ^199^ ^]^ In agriculture, precise temperature data allows farmers to optimize planting schedules for crops such as wheat and corn, which have narrow temperature thresholds for germination and growth, while also influencing crop phenology, pest outbreaks, and soil health.^[^ ^200^ ^]^ Accurate monitoring enhances agricultural productivity and mitigates risks posed by heatwaves that threaten crop survival.^[^ ^201^ ^]^ In marine environments, temperature monitoring is vital for understanding the dynamics of aquatic ecosystems, predicting changes in marine biodiversity, and managing the health of coral reefs, fisheries, and other sensitive habitats.^[^ ^202^ ^]^ In natural ecosystems such as forests, continuous monitoring of air temperature is vital for preserving biodiversity and maintaining ecological balance, as even slight shifts can disrupt species distributions, seasonal behaviors, and habitat suitability for temperature‐sensitive organisms.^[^ ^199^, ^200^ ^]^

LCAs of printed temperature sensors indicate that manufacturing energy and substrate materials are the dominant impact contributors, accounting for over 70 percent of total cradle‐to‐gate environmental burdens, while metal‐based conductive inks such as silver or indium compounds contribute disproportionately to resource depletion and human toxicity categories.^[^ ^203^ ^]^ By replacing these components with biodegradable substrates and carbon‐based inks, (bio)degradable temperature sensors significantly reduce embodied emissions and resource intensity.^[^ ^203^ ^]^ Their operational lifespan of ≈8 to 12 weeks aligns with seasonal crop cycles and short‐term ecological observations, allowing complete functionality within the intended monitoring period without requiring retrieval.^[^ ^172^ ^]^ Consequently, these transient devices provide a low‐impact, sustainable alternative to conventional non‐degradable temperature sensors that are energy‐intensive to manufacture and environmentally persistent post‐deployments. The fully and partially biodegradable temperature sensors discussed in this section are integrated with non‐biodegradable readout systems for signal acquisition and data acquisition.

**Figure 7:**
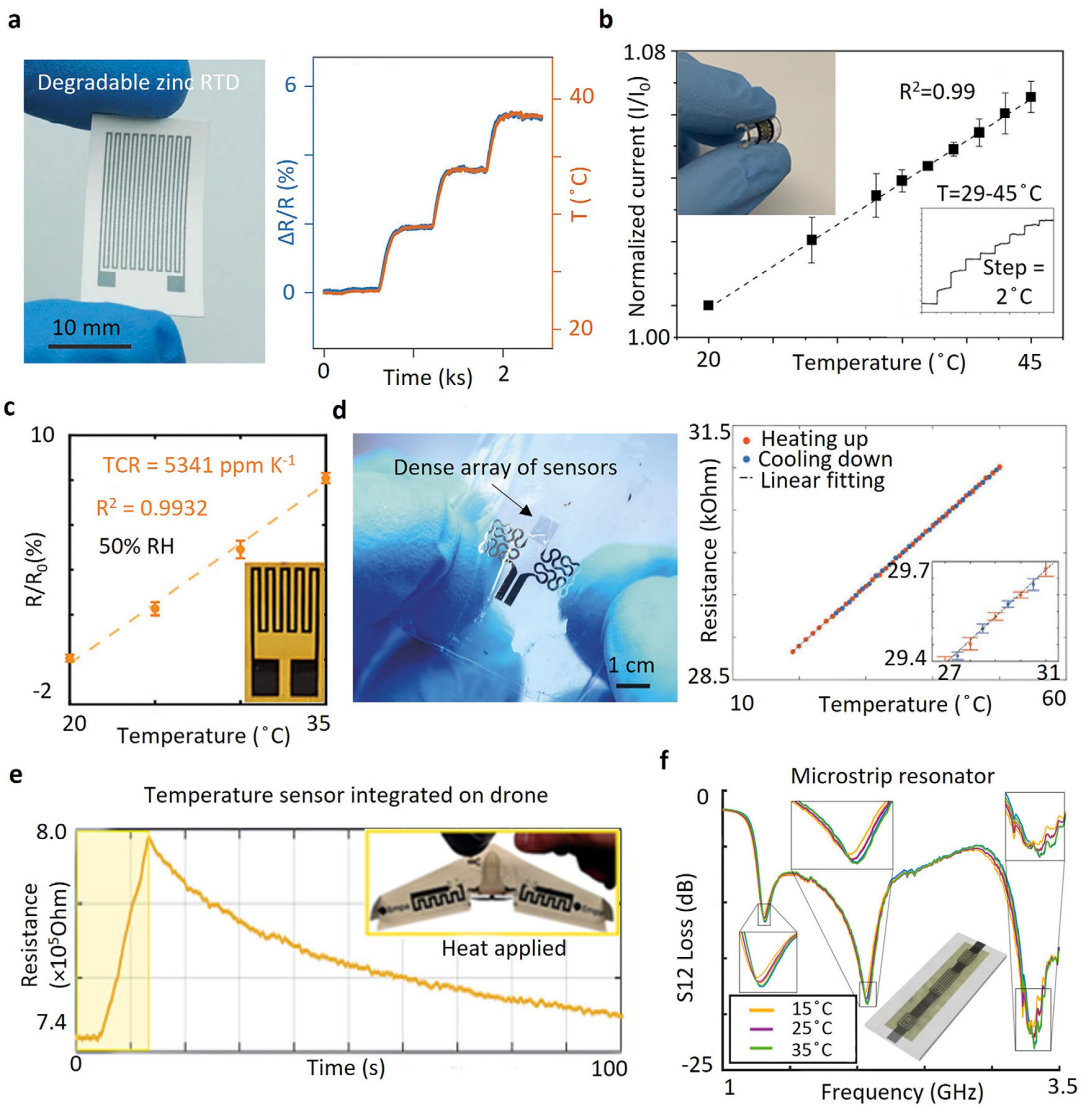
Degradable sensors for environmental temperature monitoring: a) Biodegradable screen‐printed Zn electrode on a cellulosic substrate as a temperature sensor. Reproduced with permission.^[^ ^45^ ^]^ Copyright 2023, Elsevier. b) Partially biodegradable screen‐printed silk‐based flexible thermal sensor with a linear response between 20–45 °C. Reproduced with permission.^[^ ^51^ ^]^ Copyright 2021, American Chemical Society. c) Shellac/carbon‐based temperature sensor with the linear response of relative resistance variations at 50% RH. Reproduced with permission.^[^ ^43^ ^]^ Copyright 2022, Wiley. d) Miniaturized cleanroom‐fabricated partially biodegradable sensor with linear response within 20–50 °C for temperature sensing in water medium (scale bar: 2 cm). Reproduced with permission.^[^ ^42^ ^]^ Copyright 2017, Wiley. e) Resistive screen‐printed temperature sensor patterned on the glider's wing for outdoor temperature monitoring. Reproduced with permission.^[^ ^210^ ^]^ Copyright 2023, Wiley. f) Ecoresorbable multiresonator screen‐printed sensor for temperature monitoring within the range of 15–35 °C. Reproduced with permission.^[^ ^211^ ^]^ Copyright 2024, Multidisciplinary Digital Publishing Institute. All degradable sensors shown are integrated with non‐biodegradable readout systems for signal processing and measurement.

Biodegradable sensors are emerging as sustainable solutions for temperature monitoring in agriculture. The partially biodegradable sensor introduced by Fumeaux et al.,^[^ ^45^ ^]^ illustrated in **Figure** **7**a, is capable of detecting temperatures up to 40 °C, with performance comparable to commercial sensors. However, its reliance on beeswax encapsulation introduces durability challenges in high‐temperature or high‐humidity environments, such as those exceeding 50 °C or 90% RH. The device requires supporting electronics for resistance readout. The temperature response is governed by the temperature coefficient of resistance (TCR), which describes how a material's electrical resistance changes with temperature—higher TCR values typically indicate greater sensitivity. This sensor exhibits a TCR value of 3160 ppm K^−1^, demonstrating strong temperature sensitivity while maintaining biodegradability.

Targeted for in‐soil applications, the degradable temperature sensor developed by Pradhan et al.,^[^ ^51^ ^]^ featuring screen‐printed carbon electrodes on a silk‐based substrate, exhibits high sensitivity (0.99%/°C) within a 20–45 °C range and stability under 90% RH (as shown in Figure 7b). However, its narrow operational range limits its use in industrial or high‐temperature agricultural applications, where monitoring over 80–110 °C is required. Enzymatic degradation tests conducted in vitro using a 3.5 U mL^−1^ protease solution at 37 °C showed that the silk substrate began to lose structural integrity within a few days and completely disintegrated after ≈10 days. During this period, sensing reliability decreased significantly once surface cracking and delamination appeared, typically after the initial few days of enzymatic exposure. Under milder conditions (1 U mL^−1^ protease), degradation extended to approximately one month, suggesting longer operational stability. A degradable resistive temperature sensor based on inkjet printing of carbon, as reported by Bartnik et al.,^[^ ^204^ ^]^ exhibited a high degree of linearity over a range of 30–110 °C, with a TCR of 13.2 × 10^−4^ K^−1^ and a sensitivity of 85.85 Ω °C^−1^, outperforming the silk‐based sensor in both sensitivity and range.

The partially biodegradable resistive sensor illustrated by Aeby et al.^[^ ^43^ ^]^ (see Figure 7c) shows promise for soil environments, offering a linear response to temperature changes between 20–35 °C and a TCR of 5341 ppm K^−1^. However, the temperature sensitivity of such devices can be affected by environmental humidity, which introduces interference in resistive measurements. To mitigate this issue, Aeby et al. employed a shellac substrate, reported to be nearly insensitive to humidity, providing stability comparable to glass and superior to paper‐based alternatives.^[^ ^205^ ^]^ Beyond this, other approaches to achieve humidity insensitivity in biodegradable sensors include the use of hydrophobic biodegradable coatings such as polyanhydrides and Ecoflex, applied as thin conformal or micropatterned layers.^[^ ^87^, ^206^, ^207^ ^]^ Additionally, micropillar array structures based on poly(l‐lactide‐*co*‐ε‐caprolactone) (PLCL), a biodegradable elastomer, increase surface roughness, offering a superhydrophobic surface that blocks moisture uptake, thereby minimizing humidity interference.^[^ ^208^ ^]^ Degradation tests under aerobic composting confirmed that the sensors lost ≈84.5% of their mass within 77 days, demonstrating their compatibility with compost disposal. Visible signs of degradation appeared after the first week, indicating that sensor readings may not remain reliable after this point.

Marine environmental monitoring requires sustainable and resilient sensing technologies. The partially biodegradable, flexible, and stretchable temperature sensor developed by Salvatore et al.^[^ ^42^ ^]^ (Figure 7d), utilizing Mg resistive lines and Ecoflex layers, is particularly suited for this purpose. Its sensitivity of 70 Ω K^−1^ over a 20–50 °C range makes it effective for monitoring temperature fluctuations in aquatic settings while minimizing ecological impact. The 16 µm Ecoflex encapsulation delays complete structural dissolution to ≈67 days in saline conditions at 25 °C, yet stable electrical performance is sustained for only ≈1 day; beyond this, Mg corrosion and partial delamination cause signal drift and unreliable readings.^[^ ^42^, ^209^ ^]^

Air temperature monitoring over forest ecosystems presents unique challenges, which was beeswax encapsulation (Figure 7e), it offers a novel solution for autonomous environmental monitoring. The system operates reliably within 20–50 °C, enabling efficient data collection during flight. addressed by Kovac et al.’s^[^ ^210^ ^]^ partially transient robot system. Resistive temperature sensors, as commonly discussed so far, depend on wired readout circuits that are not biodegradable, limiting their suitability for fully transient systems. Passive resonator‐based sensors offer a compelling alternative, enabling wireless temperature sensing without non‐biodegradable electronic components. The partially biodegradable microstrip resonator sensor based on patterned Zn on a paper substrate encapsulated in beeswax, as shown in Figure 7f, exhibits a linear sensitivity of −1.35 MHz °C^−1^, making it effective for precise thermal monitoring.^[^ ^211^ ^]^ However, excessive thickness of the beeswax encapsulation can reduce sensor performance, while thinner layers mitigate this issue.^[^ ^212^ ^]^

### 5.5 Photodetectors

Photodetectors play a pivotal role in environmental monitoring by converting light signals into electrical outputs that reveal essential information about atmospheric, aquatic, and terrestrial conditions.^[^ ^213^ ^]^ Variations in light intensity and spectral composition are closely linked to environmental hazards such as ozone depletion, UV‐induced ecosystem damage, air pollution scattering, and climate‐driven changes in solar radiation balance.^[^ ^214^, ^215^ ^]^ With their high sensitivity, fast response, and broad spectral operability, photodetectors enable precise detection of pollutants, greenhouse gases, and particulate matter.^[^ ^216^, ^217^, ^218^ ^]^

LCAs of thin‐film optical and optoelectronic devices analogous to photodetectors indicate that semiconductor and electrode layers account for more than 80 percent of total climate and resource depletion impacts, primarily due to energy‐intensive deposition processes and the use of scarce metals such as indium, gallium, and silver; quantum dot synthesis further adds significant energy and solvent burdens to the overall footprint.^[^ ^219^, ^220^ ^]^ In contrast, (bio)degradable photodetectors using organic semiconductors, cellulose substrates, and carbon electrodes markedly lower embodied emissions by avoiding high‐temperature processing and critical metals.^[^ ^221^ ^]^ Their short‐term lifespan, matching seasonal environmental and ecological monitoring cycles, removes the need for retrieval and prevents long‐term waste accumulation.^[^ ^222^ ^]^

**Figure 8:**
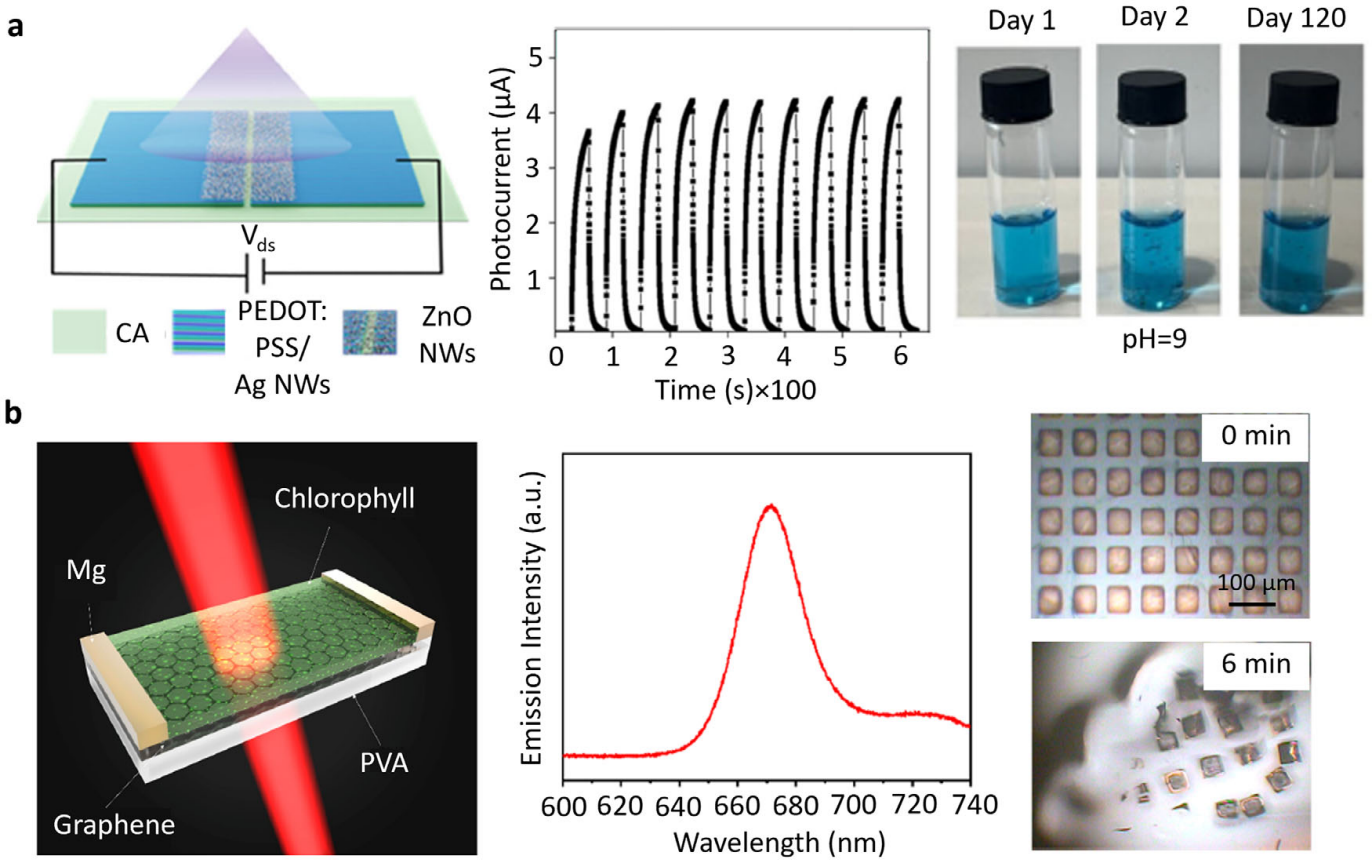
Degradable photodetectors for environmental monitoring: a) Schematic of the partially degradable photodetector based on slectrospun ZnO NWs on cellulose acetate (CA) substrate showing cyclic stability at a bias voltage of 5 V and degradation behavior over time in an aqueous environment. Reproduced with permission.^[^ ^223^ ^]^ Copyright 2025, Springer Nature. b) Schematic of the degradable cleanroom‐fabricated photodetector based on chlorophyll, showing the photoluminescence spectrum of chlorophyll and the device degradation behavior in aqueous solution. Reproduced with permission.^[^ ^224^ ^]^ Copyright 2018, American Chemical Society.

In this context, Karagiorgis et al.^[^ ^223^ ^]^ demonstrated a partially degradable and transparent photodetector fabricated from conductive poly(3,4‐ethylenedioxythiophene):polystyrene sulfonate (PEDOT:PSS):Ag nanowire‐based nanofibres and ZnO nanowires on a transparent cellulose acetate (CA) substrate, as shown in **Figure** **8**a. The device exhibited a high responsivity of 1.10 × 10^6^ A W^−1^ under dynamic UV exposure, maintaining consistent performance on both flat and curved surfaces. The reproducibility of the photocurrent was confirmed over 10 cycles of illumination at a constant light intensity of 0.1 µW cm^−2^, demonstrating stable and repeatable operation. In aqueous media, visible suspended particulate was observed after the first day, indicating the onset of material degradation. The CA substrate dissolved within four weeks, while residual PEDOT:PSS:Ag traces persisted up to 24 weeks; Although electrical performance was not reported during degradation, progressive delamination and interface weakening would likely induce photocurrent drift and reduce measurement reliability over time.

Lin et al.^[^ ^224^ ^]^ reported an eco‐friendly, degradable photodetector based on a hybrid of graphene and chlorophyll deposited on a PVA substrate, as shown in Figure 8b. The device exhibited a photoresponsivity of ≈200 A W^−1^ under ambient conditions and demonstrated complete physical dissolution in aqueous solution within ≈30 min, highlighting its transient nature. The photoluminescence spectrum of chlorophyll, obtained using a 266 nm Nd:YAG pulsed laser as the excitation source, confirmed its strong optical response. In an aqueous environment, the PVA substrate began shrinking after 2 min, followed by electrode edge contraction at 4 min and partial detachment shortly thereafter. These structural changes indicate that sensor readings are likely unreliable beyond the first few minutes of immersion due to rapid substrate deformation and electrode delamination.

The study by Karagiorgis et al. demonstrated superior photodetecting performance owing to efficient charge transfer between inorganic ZnO nanowires and conductive (PEDOT:PSS):Ag nanofibre electrodes. In contrast, Lin et al. observed lower photoresponse due to the limited carrier mobility of the chlorophyll‐based active layer. This reduction in performance, however, was offset by the device's degradation. Further examples of such degradable photodetectors have been reported; however, the next crucial step is conducting field trials to better understand their real‐world limitations and reliability under practical environmental conditions.

The selection between (bio)degradable and hybrid monitoring systems (including sensor nodes, readout, and communication modules) should be guided by the intended application, operational duration, recovery feasibility, and environmental context. Fully transient systems are typically designed for short‐term deployments lasting from a few days up to several months, after which all components naturally degrade in situ. Such configurations are ideal for remote or ecologically sensitive environments where retrieval is impractical. Hybrid systems, by contrast, integrate transient sensing modules with reusable or recyclable electronic units, supporting extended operation from several months to years. LCA studies have shown that hybrid or recyclable designs can reduce environmental impact by up to 50–60% compared with fully non‐degradable electronics, primarily through material recovery and reuse of high‐impact components such as integrated circuits and communication modules.^[^ ^142^, ^156^, ^225^, ^226^ ^]^ While comparisons with non‐degradable sensors are included in this review, they serve only to provide design inspiration and/or optimization principles from non‐degradable systems. These insights reveal architectural, interfacial, and system‐level strategies that can be adapted to enhance (bio)degradable sensor performance in future applications. The focus remains on the steady progress within (bio)degradable technologies, where advances in materials, device design, and integration have already yielded measurable functional improvements. Ultimately, the system configuration should balance functionality, operational lifetime, and recovery feasibility rather than aim to replicate the performance of traditional silicon‐based devices.

Fully transient systems, in which all components degrade after use, are best suited for temporary monitoring in environments where device recollection is not feasible. For example, (bio)degradable sensors for biodiversity monitoring in dense forest ecosystems can be dispersed using aerial drones. These sensors can operate passively based on resonant elements through backscattering communication, where a remote station transmits a carrier signal that is modulated and reflected by (bio)degradable coils or antennas integrated within the sensor. The reflected signal is demodulated to extract sensing data, enabling autonomous operation without onboard non‐degradable electronics.

Hybrid systems combining (bio)degradable sensors with reusable or recyclable electronics are more appropriate for long‐term or data‐intensive applications requiring greater signal fidelity, amplification, or extended communication range.^[^ ^156^, ^227^ ^]^ Complementary metal–oxide–semiconductor (CMOS)‐based front‐end circuits can perform excitation, measurement, and processing, while wireless modules such as Wi‐Fi, Bluetooth Low Energy (BLE), or Long Range (LoRa) transceivers enable real‐time telemetry and cloud‐based data management.^[^ ^41^, ^156^, ^172^ ^]^ Multiplexed readout circuits allow multiple sensors to share acquisition channels, improving scalability and power efficiency. Depending on circuit demands, hybrid systems can also be powered by (bio)degradable thin‐film batteries, supercapacitors, or solar harvesters, as discussed in Section 7.

A key advantage of hybrid configurations is the potential to retrieve and reuse non‐transient components once the degradable elements have disintegrated. Retrieval can be achieved using autonomous drones or ground rovers equipped with magnetic or mechanical grippers.^[^ ^228^, ^229^ ^]^ Recovered modules can be cleaned, tested, and redeployed, while irreparable parts are directed to certified electronic recycling streams for material recovery of high‐value metals such as Ag and safe processing of residual components, including solder alloys, ceramics, and polymer encapsulants.^[^ ^230^ ^]^ Biodegradable encapsulants or substrates can be composted or degraded as discussed in the degradation section, based on the environment. This closed‐loop approach supports circular‐economy principles by extending component lifespan and minimizing waste.^[^ ^226^ ^]^

Integrating (bio)degradable CMOS microcontrollers with degradable power sources represents a major step toward transient readout and communication systems that combine sustainability with enhanced functionality. Early work by the Rogers group demonstrated the first transient CMOS inverter using ultrathin monocrystalline silicon nanomembranes as semiconductors, Mg interconnects, and SiO_2_ dielectrics on PLA substrate.^[^ ^227^ ^]^ The inverter achieved a voltage gain of ≈50 and a threshold voltage (Vth) near −1 V at drain supply voltage (Vdd) = 10 V, confirming that reliable logic operation is possible with transient materials. Ongoing research aims to reduce silicon thickness to a few micrometers to shorten device lifetime while maintaining performance uniformity. Conventional CMOS devices, however, rely on non‐degradable encapsulants such as epoxy mold compounds, polyimides, silicones, and underfill resins that release hazardous byproducts during disposal.^[^ ^226^ ^]^ A promising route toward sustainable integration involves compostable circuit substrates and green printed circuit boards (PCBs) that replace conventional FR4 with bio‐based cellulose materials, which combine mechanical robustness with biodegradability.^[^ ^231^, ^232^, ^233^ ^]^ Future work should focus on improving these materials for scalable manufacturing, barrier performance, and CMOS compatibility while maintaining material loads below ecosystem‐specific toxicity thresholds.

Overall, recent progress demonstrates that environmentally friendly electronics can be achieved through thoughtful system design rather than material substitution alone. Short‐term transient devices provide an elegant solution for applications where recollection is impractical, while hybrid systems balance durability with recoverability and efficient data handling. Emerging biodegradable semiconductors, cellulose‐based substrates, and closed‐loop recovery methods are redefining how electronic monitoring networks can operate within the boundaries of a circular economy. **Table** **4** summarizes and compares the advantages and disadvantages of biodegradable, partially biodegradable, and nondegradable sensors, highlighting their performance and alignment with the United Nations Sustainable Development Goals (SDGs).

**Table 4:** Comparative overview of biodegradable, partially biodegradable, and nondegradable sensors for environmental and ecological monitoring.

Sensor Type	Application	Sensing mechanism	Sensitivity / Responsivity	CO_2_e (kg per unit)	Stability / Drift	Advantages / Limitations and considerations	Environmental impact and SDG^a)^ alignment	References
Biodegradable	Moisture/humidity/ temperature	Resistive / Capacitive	9 MHz/%RH; 0.8–1.0%/°C	0.025–0.04 kg CO_2_e (25–40 g) → ≈93–96% lower than nondegradable	±5–15% drift after 1–3 weeks; degradation under high RH/heat	Fully transient systems designed for mass, one‐shot deployments without retrieval. Enable distributed sensing via aerial/terrestrial robots. Advantages: ultra‐low CO_2_ footprint, compostable materials, compatible with green and scalable fabrication, zero e‐waste. Limitations: considerable drift while degradation, limited stability under high humidity, and a need for protective encapsulation tuned for the target sensing mechanism and applicable environment	Fully compostable; zero e‐waste. SDGs: 6, 12, 13, 14–15	[13, 32, 74, 145, 234, 235]
	Light / UV	Optical (Photodetector)	∼200 A/W		Stable for minutes–hours; drift increases with substrate delamination			[224, 234, 235]
	pH/chemical	Electrochemical	LOD ≈ 0.009–500 µM (application‐dependent)		±10–20% drift after 1–2 weeks due to electrode degradation			[21, 48, 50, 174, 234, 235]
Partially Biodegradable	Moisture/humidity /temperature	Resistive / Capacitive / Microstrip resonator	TCR ≈ (3–5)×10^3^ ppm/K; 85.85 Ω/°C; 70 Ω/K	0.05–0.2 kg CO_2_e (∼60–80% lower than nondegradable)	±2–10% drift; oxidation‐related decay	Hybrid systems combining transient sensing layers with retrievable or recyclable electronics. Advantages: reduced CO_2_ emissions; partial biodegradability; possibility of recovering and reusing non‐degradable modules; compatible with scalable and green roll‐to‐roll fabrication. Limitations: incomplete degradation; potential toxic metallic residues; oxidation drift; encapsulation required for stability.	Supports circular economy, material recovery. SDGs: 9, 12, 13, 15	[42, 43, 44, 45, 87, 142, 146, 204, 205, 226, 234, 235]
			−0.8 to −8 MHz/%RH; −1.35 MHz/°C		±5–10% drift due to Zn oxidation			[153, 154, 211, 212, 235]
	Light / UV	Optical (Photodetector)	∼10^5^–10^6^ A/W		Stable ≈ 10 days; delamination after weeks			[223, 234, 235]
	pH/chemical	Electrochemical	LOD ≈ nm–µm		±10% drift after 2–4 weeks			[39, 148, 149, 175, 177, 235]
Nondegradable	Moisture/humidity	Capacitive / Resistive	±1–2% RH; < 5 s response	≈ 0.6 kg CO_2_e (≈ 613 g)	< ±1% drift over > 5 years	Nondegradable systems optimized for precision and longevity. Advantages: mature technology, high accuracy, multi‐year reliability, broad operational range. Limitations: persistence >10s of years, Non‐green fabrication, energy‐intensive recycling, high CO_2_ impact, unsuitable for large‐scale disposable use.	High CO_2_ emissions; persistent pollution; non‐recyclable. Conflicts with SDGs: 12, 13, 14–15	[13, 146, 178, 235]
	pH/chemical	ISFET	55–60 mV/pH (linear)		< ±0.5% drift/month (with calibration)			[161, 162, 235]
	Temperature	Resistive	TCR > 5×10^3^ ppm/K; ±0.1 °C precision		< ±0.1 °C drift per year			[204, 210, 235]
	Chemical	CMOS	LOD ≈ ppb–ppm; response < 1 s		Stable months–years; minor drift			[178, 179, 187, 235]
	Light / UV	Optical (Si, GaAs photodiodes)	Responsivity ∼0.1–0.6 A/W; response < 1 ms–ms		Negligible‐to‐low drift over lifetime			[235, 236, 237]

^a)^ SDG: Sustainable Development Goals (based on the United Nations 2030 Agenda for Sustainable Development). Relevant goals include SDG 6 (Clean Water and Sanitation), SDG 9 (Industry, Innovation, and Infrastructure), SDG 12 (Responsible Consumption and Production), SDG 13 (Climate Action), SDG 14 (Life Below Water), and SDG 15 (Life on Land).^[^ ^235^ ^]^

## 6 Manufacturing Techniques for Biodegradable Electronics Dedicated to Environmental Applications

Fabrication plays a critical role in the performance and longevity of ecoresorbable devices, as biodegradable materials are inherently sensitive to processing conditions. Their key properties, such as hydrophilicity, crystallinity, and degradation kinetics, can be easily altered during manufacturing. Without precise control, these characteristics may be compromised, leading to reduced functionality or premature degradation. For example, hydrophilic polymers like PEG and PVA are prone to hydrolytic degradation, necessitating strict regulation of chemical exposure and thermal conditions during fabrication to maintain device integrity.^[^ ^19^, ^238^, ^239^ ^]^

A key challenge in fabricating biodegradable devices is the limited temperature tolerance of many biopolymers, which restricts the use of conventional high‐temperature processes. This makes low‐temperature methods such as stencil lithography and printing especially attractive for patterning sensitive substrates while maintaining material integrity.^[^ ^240^ ^]^

In addition to preserving material properties, fabrication strategies must also align with broader sustainability goals. As the environmental footprint of production becomes increasingly relevant, integrating eco‐friendly approaches into manufacturing processes is essential. Sustainable practices, including solvent recycling and energy‐efficient methods, help reduce waste and emissions. Solvent‐free techniques, such as supercritical fluid extraction and heat‐pressure‐based bonding, not only improve product purity but also eliminate the need for energy‐intensive purification steps.^[^ ^241^, ^242^, ^243^, ^244^, ^245^ ^]^ The use of renewable energy sources ‐such as solar and wind power‐ and advanced technologies like low‐temperature plasma treatments contribute to lowering carbon footprints and minimizing ecological harm. The following section provides an overview of fabrication methods commonly employed for biodegradable devices used in environmental monitoring.

### 6.1 Printing

Printing encompasses a broad range of additive manufacturing techniques, including screen, 3D/4D printing, and electroplating. Among these, screen printing is the most established and widely used for fabricating degradable environmental sensors, due to its scalability, simplicity, and compatibility.^[^ ^93^ ^]^ Recent studies on a 3D‐printed humidity sensor using PLA ink and electroplating‐assisted additive processes for Zn electrodes further highlight the expanding potential of printing technologies in sustainable device fabrication.^[^ ^246^, ^247^ ^]^

Screen printing involves transferring ink or functional materials through a mesh screen onto a substrate to create precise patterns or layers. This technique offers scalability, cost‐effectiveness, and the ability to deposit thick, high‐resolution layers on diverse substrates. While it is versatile and widely used in electrode fabrication, screen printing has limitations, such as lower resolution compared to lithography and potentially longer production times due to the need for multiple passes to achieve thick layers.^[^ ^248^, ^249^ ^]^

**Figure 9:**
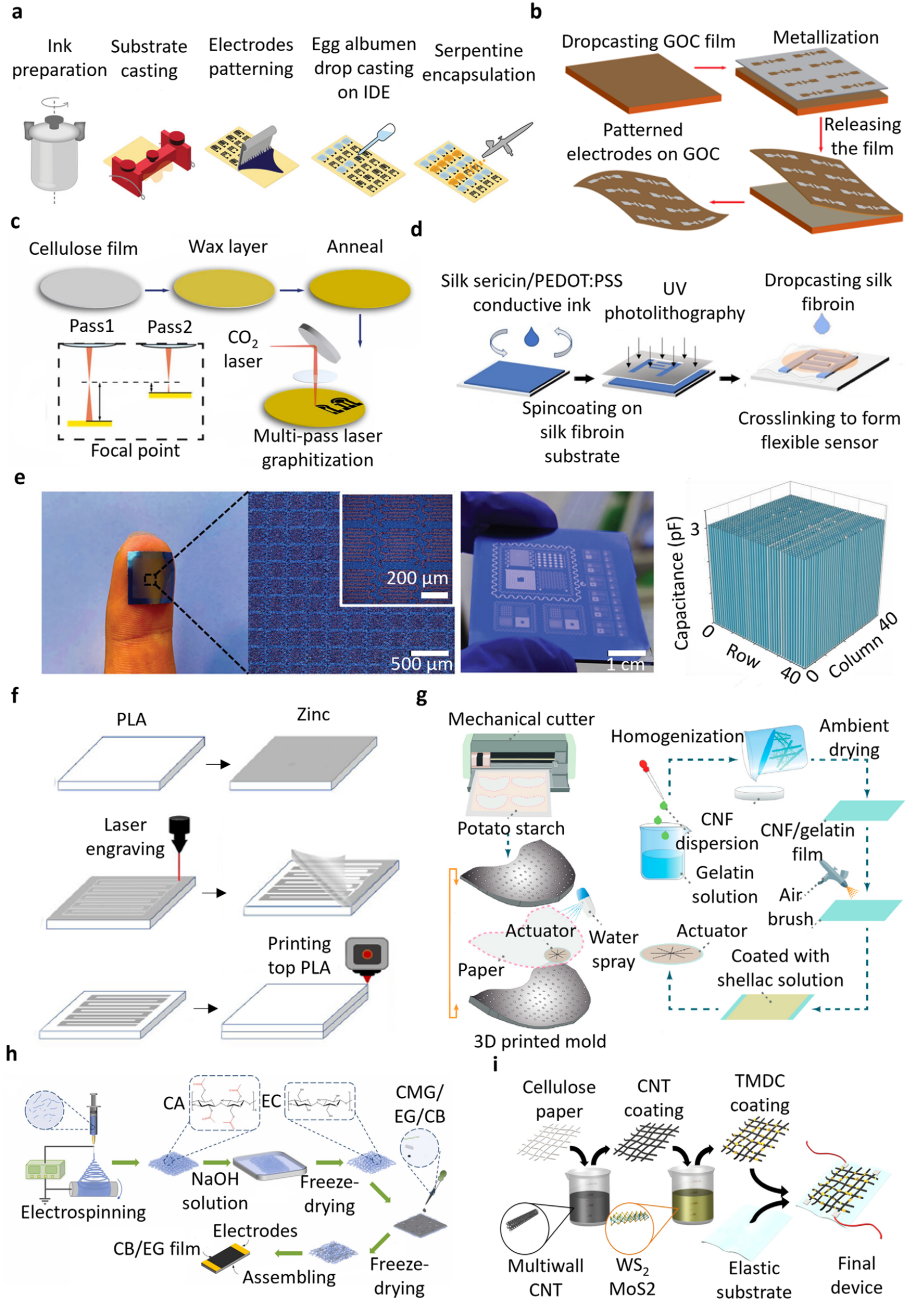
Fabrication methods utilized for bio‐disintegrable devices: a) Fabrication of the temperature and RH sensors based on screen printing of the carbon ink. Reproduced with permission.^[^ ^43^ ^]^ Copyright 2022, Wiley. b) Fabrication of a resistive sensor by metal patterning using shadow mask technique. Reproduced with permission.^[^ ^289^ ^]^ Copyright 2020, Institute of Electrical and Electronics Engineers (IEEE). c) Laser‐induced graphitization method for electrode fabrication directly on the substrate. Reproduced with permission.^[^ ^50^ ^]^ Copyright 2023, Elsevier. d) Schematic of the formation of a flexible silk/poly(3,4‐ethylenedioxythiophene):polystyrene sulfonate (PEDOT:PSS) electrodes with UV cross‐linking through a hard mask. Reproduced with permission.^[^ ^51^ ^]^ Copyright 2020, American Chemical Society. e) Microfabrication of highly miniaturized and partially biodegradable devices on PLA with consistent performance based on photolithography, thin‐film deposition, reactive ion etching, and lift‐off. Reproduced with permission.^[^ ^19^ ^]^ Copyright 2023, Wiley. f) Manufacturing of chipless sensors by a combination of 3D printing of PLA followed by the laser cutting of Zn layer. Reproduced with permission.^[^ ^32^ ^]^ Copyright 2022, Springer Nature. g) Step‐by‐step fabrication of biodegradable glider, incorporating mechanical cutting, solution casting and spray coating methods. Reproduced with permission.^[^ ^21^ ^]^ Copyright 2022, Frontiers. h) Fabrication steps of conductive droplet‐based VOC sensor based on electrospinning and freeze‐drying. Reproduced with permission.^[^ ^44^ ^]^ Copyright 2024, Elsevier. i) Dip coating method for fabrication of carbon/cellulose electrodes for a partially biodegradable chemical sensor.^[^ ^184^ ^]^ Reproduced with permission. Copyright 2019, American Chemical Society.

The humidity sensor developed by Aeby et al.,^[^ ^43^ ^]^ as illustrated in **Figure** **9**a, exemplifies the potential of screen printing for sustainable device fabrication. Made from sustainable materials like shellac and cellulose, the sensor reduces environmental impact by avoiding synthetic polymers and non‐biodegradable components while functioning as both RH and temperature sensors. Its compact design, with a 1 cm^2^ footprint, further highlights its suitability for miniaturized applications. It reduces production costs by up to 50% compared to photolithography while maintaining compatibility with biodegradable substrates, supporting sustainable manufacturing practices.^[^ ^250^, ^251^ ^]^ However, challenges such as variability in electrode uniformity and surface roughness remain, potentially affecting precision and durability in high‐performance applications.^[^ ^43^ ^]^

Screen printing can also evolve into an environmentally friendly process by adopting water‐based inks, such as those derived from natural pigments, which significantly reduce the release of volatile organic compounds (VOCs) compared to conventional solvent‐based inks. Depending on the formulation, these inks allow precise layer formation, typically ranging from 30 to 50 µm.^[^ ^252^ ^]^ For example, modified water‐based acrylic emulsions have shown improved environmental performance by reducing VOC emissions from 3373 ppm (solvent‐based inks) to 2478 ppm.^[^ ^252^ ^]^ Advancements in water‐based formulations, such as graphene oxide and silver nanowire inks, further enhance the capabilities of screen printing. Graphene oxide inks enable thin layers below 6 µm with low sheet resistance, suitable for flexible substrates.^[^ ^253^ ^]^ Silver‐nanowire inks achieve high conductivity (≈4.67 × 10^4^ S cm^−1^).^[^ ^254^ ^]^ However, silver nanowires is non‐biodegradable, and this highlights the need to develop fully water‐based, biodegradable conductive ink alternatives.

The screen‐printing method employed by Aeby et al. reached electrode gap sizes of 200 µm, with surface roughness levels similar to those observed in conventional printed electronic devices.^[^ ^43^ ^]^ Comparatively, studies such as Zhang et al.^[^ ^255^ ^]^ reported that screen printing with carbon nanotubes (CNT)‐based inks achieved layer thicknesses below 6 µm, providing greater stability and conductivity for flexible devices. However, CNTs are non‐biodegradable and known to pose environmental toxicity concerns.^[^ ^256^ ^]^

To address these challenges, optimizing ink rheology and mesh design can improve printing precision and uniformity. Hybrid fabrication approaches, such as combining screen printing with inkjet deposition, laser patterning, or photolithography, enable higher resolution and smoother surfaces than conventional screen printing. In particular, inkjet–photolithography integration enables localized ink deposition within photo‐patterned regions, followed by selective curing or etching to define precise geometries. Through controlled droplet placement and layer‐by‐layer photopolymerization or removal, the process achieves high‐aspect‐ratio (>3:1) microstructures with alignment accuracy below 10 µm, enabling reliable fabrication of next‐generation miniaturized and flexible electronic devices.^[^ ^257^ ^]^

### 6.2 Shadow Mask Patterning

Shadow mask patterning is a simple, solvent‐free technique well‐suited for biodegradable and flexible substrates, offering advantages over lithography such as low thermal and chemical stress and reduced chemical waste. It eliminates photoresists and harsh etchants, preserving sensitive materials like PLA, cellulose, and silk fibroin.^[^ ^258^, ^259^, ^260^, ^261^ ^]^

The fabrication of the resistive sensor shown in Figure 9b using the shadow mask technique highlights both the advantages of simplicity and scalability, as well as the limitations in resolution inherent to this method.^[^ ^50^ ^]^ The method achieves a precise 60 µm gap between electrodes, ensuring consistent patterning across the substrate. Its straightforward nature makes it attractive for small‐scale fabrication; however, the reliance on sacrificial layers, such as cellulose acetate butyrate, introduces environmental concerns. The dissolution process using acetone generates chemical waste and limits eco‐friendliness, which could pose challenges in terms of sustainability. Aligning the shadow mask accurately is a meticulous process, with even slight misalignments compromising the device's performance and reproducibility—issues that are especially critical in mass production.^[^ ^262^ ^]^

Another approach using shadow masks in optical filter fabrication achieved spatial variations controlled at ≈50 µm resolution through evaporation, significantly enhancing the uniformity and optical performance of filters.^[^ ^263^ ^]^ Using reusable Si masks, pattern resolutions of ≈100 nm have been achieved on polymeric substrates, suitable for biodegradable and flexible sensors.^[^ ^264^ ^]^

Despite these strengths, challenges remain with shadow mask techniques, such as achieving finer resolutions and maintaining alignment precision during mass production. For instance, the degradation of shadow masks due to repeated use and warping can reduce accuracy, although cleaning procedures can restore functionality.^[^ ^260^ ^]^ Advanced hybrid techniques, such as combining shadow mask deposition with nanoscale lithography, have resulted in improved resolutions, with methods like azimuthal and polar angle‐resolved shadow mask deposition combined with nanosphere lithography achieving patterns down to 50 nm while maintaining scalability and uniformity over large areas.^[^ ^265^ ^]^ These limitations highlight the need for further innovations, such as integrating machine‐assisted alignment systems or adopting hybrid deposition techniques.

### 6.3 Laser‐Assisted Material Formation

Laser‐assisted material formation (LAMF) is a key technique for fabricating functional layers, enabling precise control over material properties and structures. Using a high‐energy laser beam, LAMF allows for deposition, ablation, or modification with high spatial resolution, which is essential for complex functional layers in diverse applications.^[^ ^266^, ^267^ ^]^ CO_2_ lasers are particularly effective for processing organic materials and polymers due to their long wavelength (10.6 µm), while Nd:YAG lasers are suited for metals and excimer lasers are employed for semiconductor etching.^[^ ^268^ ^]^ UV lasers are commonly applied in surface patterning and photochemical cross‐linking,^[^ ^51^ ^]^ and femtosecond lasers are ideal for micro‐ and nanoscale structuring.^[^ ^238^ ^]^ Despite its versatility, LAMF faces challenges, including material compatibility, processing speed, and cost‐effectiveness when scaling for larger applications.^[^ ^266^, ^269^ ^]^

A notable subset of LAMF is laser‐assisted carbonization, which focuses on converting carbon‐rich biodegradable substrates—such as cellulose—into conductive carbon structures. Unlike general LAMF processes that involve material addition or removal, carbonization drives graphitization through localized thermal decomposition, resulting in carbon‐based electrodes that have been reported to be biodegradable.^[^ ^270^, ^271^, ^272^ ^]^ As illustrated in Figure 9c, CO_2_ laser‐induced carbonization of cellulose forms flexible, conductive electrodes with a sheet resistance of 43.7 ± 2.3 Ω sq^−1^.^[^ ^50^ ^]^ This process eliminates the need for harsh chemicals or high‐temperature treatments, reducing energy consumption and environmental impact. Utilizing wood and other renewable resources supports sustainability and minimizes waste.^[^ ^266^, ^267^ ^]^

In terms of performance, CO_2_ laser‐induced graphitization has shown promising conductivity for biodegradable electrodes. A distinct subset of this process, LIG, uses optimized laser conditions to convert carbon‐rich substrates into porous networks enriched with sp^2^‐hybridized, graphene‐like domains.^[^ ^273^ ^]^ For instance, LIG fabricated on paper can achieve sheet resistances as low as 16 Ω·sq^−1^ under optimized conditions.^[^ ^272^, ^274^ ^]^ Integrated LIG electrodes in microfluidic devices feature high‐resolution patterns and maintain consistent sheet resistances of 71 ± 15 Ω sq^−1^.^[^ ^93^, ^275^ ^]^ While these values indicate moderate conductivity, the performance of LIG—particularly sheet resistances as low as 16 Ω·sq^−1^—is approaching that of silver nanowire films, which remain the benchmark despite silver's non‐biodegradable nature.^[^ ^276^ ^]^

Another promising LAMF direction is UV laser cross‐linking, as illustrated in Figure 8d, where Pradhan et al.^[^ ^51^ ^]^ utilized a photoactive ink blend of silk, photosericin, and reduced graphene oxide. This method enabled precise electrode patterning via spin‐coating and UV exposure, with feature sizes as small as 2 µm, suitable for high‐resolution devices like hybrid photodetectors.^[^ ^277^ ^]^ Current limitations of this method include uneven cross‐linking over large areas and inconsistent ink performance during spin‐coating, which hinders scalability.

To address current limitations in both LIG and UV laser cross‐linking, advancements such as integrating machine learning to dynamically optimize laser parameters could significantly improve reproducibility, precision, and scalability across different substrates and patterns.^[^ ^278^ ^]^ For LIG, future research should investigate the potential of alternative biodegradable precursors beyond cellulose or wood—such as agricultural residues, biopolymers, or marine biomass—to fabricate conductive (bio)degradable electrodes.^[^ ^279^ ^]^ In the context of UV laser cross‐linking, exploring the use of degradable metal powders as conductive fillers and developing biodegradable photoactive agents—such as lignin—could expand material applicability while maintaining performance.^[^ ^280^ ^]^

### 6.4 Photolithography

Photolithography is a foundational microfabrication technique widely recognized for its precision and scalability in defining intricate patterns on substrates, making it essential in semiconductor manufacturing and advanced microelectronics. Recent advancements, such as the microfabrication strategy proposed by Bathaei et al.^[^ ^19^ ^]^ (as shown in Figure 9e), have successfully applied this method to fabricating miniaturized sensors on biodegradable substrates, achieving high‐density integration with 1600 sensors within a 1 cm^2^ footprint. Using germanium as a protective layer and SiO_2_ as an adhesion layer enabled standard clean‐room processes to pattern fine metal lines with a resolution of 10 µm, indicating high repeatability and reliability. However, the reliance on photolithography, which involves harsh chemicals, significant energy consumption, and thermal steps such as photoresist baking, introduces challenges for low–glass transition temperature (Tg) biodegradable substrates like poly(3‐hydroxybutyrate (PHB) or PCL, which may soften or deform during processing.^[^ ^281^ ^]^ These factors collectively limit the environmental sustainability and substrate compatibility of the method.

Bathaei et al.’s method distinguishes itself by achieving sensor densities similar to those of conventional Si‐based counterparts while incorporating biodegradable materials into the fabrication process.^[^ ^282^ ^]^ Compared to alternative approaches, such as dry‐transferable photoresists shown by Chen et al.,^[^ ^283^ ^]^ which offer greater environmental friendliness and compatibility with flexible electronics, Bathaei et al.’s strategy involves more complex fabrication steps and less environmental adaptability. Similarly, dry‐film photoresists, as explored by Roos et al.,^[^ ^284^ ^]^ have been shown to reduce energy consumption and toxic chemical use but may compromise resolution or scalability.^[^ ^284^ ^]^

Techniques such as solvent‐free or plasma‐free microfabrication and scalable methods like photosensitive sol‐gels or direct burn‐in patterning, as suggested by Wu et al.,^[^ ^285^ ^]^ could further enhance the viability of biodegradable sensors. Incorporating microfabrication practices that prioritize environmental sustainability into existing protocols can help reduce impact without compromising resolution or reliability.

### 6.5 Laser Ablation and Cutting

Laser ablation and cutting are versatile and direct fabrication methods extensively used in the development of (bio)degradable devices, enabling intricate shaping of materials such as (bio)degradable metals and polymers.^[^ ^16^ ^]^ This technique leverages a high‐energy laser beam to cut or engrave materials with high precision, facilitating custom‐designed components suitable for both prototyping and large‐scale production. Its advantages include high‐speed processing and compatibility with various biodegradable and renewable materials, making it a sustainable option. However, challenges such as the formation of heat‐affected zones and material limitations, particularly with thicker or heat‐sensitive substrates, can affect performance and scalability.^[^ ^286^ ^]^

Incorporating energy‐efficient lasers and using biodegradable materials can further reduce the environmental footprint. For instance, the fabrication method employed for the fully passive, wireless, and biodegradable moisture sensor combines laser ablation with a PLA substrate to achieve precise patterning and operational efficiency (see Figure 9f).^[^ ^32^ ^]^ The sensor utilizes Zn as a degradable conductive material, providing superior performance due to its high conductivity (≈10^7^ S m^−1^) and high Q‐factor at high frequencies. At a resonance frequency of 1.5 GHz, it outperforms other degradable metals like iron. Laser ablation enhances the dimensional accuracy of the patterning process, achieving resolutions of 10–20 µm.^[^ ^287^ ^]^ However, compared to femtosecond laser‐based techniques, which can achieve resolutions down to 2 µm while maintaining mechanical integrity,^[^ ^288^ ^]^ this method is less competitive for ultra‐fine patterning.

Real‐time monitoring and advanced techniques, such as magnetically assisted laser‐induced plasma ablation, can enhance precision, reproducibility, and scalability.^[^ ^290^ ^]^ Ultrashort pulsed lasers, including femtosecond and picosecond lasers, minimize heat‐affected zones, making them ideal for biodegradable materials.^[^ ^291^, ^292^ ^]^ Gigahertz (GHz) burst mode ultrashort pulsed laser ablation offers an alternative strategy to increase ablation efficiency while reducing heat accumulation, improving the quality of microfabrication in biodegradable substrates.^[^ ^293^ ^]^

### 6.6 Spin/Solution Casting and Spray Coating

Solution casting and spray coating are pivotal techniques in the fabrication of biodegradable devices, each with unique advantages and constraints. Solution casting entails dissolving biodegradable polymers in a solvent and casting the solution onto a substrate, forming a uniform film upon solvent evaporation.^[^ ^294^ ^]^ This method is noted for its simplicity and cost‐effectiveness, although it requires extended drying times and is limited by solvent‐substrate compatibility.

Conversely, spray coating atomizes the polymer solution into fine droplets, which are precisely deposited onto a substrate, enabling rapid, large‐area coverage and fine control over film thickness and morphology.^[^ ^21^ ^]^ Despite its scalability and suitability for complex geometries, spray coating results in material wastage and necessitates sophisticated equipment for uniform application. Both techniques are essential for creating functional layers in biodegradable devices, facilitating advancements in transient electronics, environmental sensors, and medical implants.

The fabrication of biodegradable glider, illustrated in Figure 9g, exemplifies the fabrication process, including solution casting and spray coating.^[^ ^21^ ^]^ A shellac layer, applied via spray coating, enhances the device's durability under environmental conditions. However, using conventional solvents and inefficient spray techniques in these processes introduces environmental concerns, such as chemical pollution and material waste, that hinder the sustainability of the fabrication method.^[^ ^295^, ^296^ ^]^ Mechanical cutting of potato starch wafer paper for the glider's wings, while precise, risks material wastage if not optimized.

A key limitation of solution casting and spray coating is their inability to directly produce patterned films, as they typically form continuous layers suited mainly for encapsulation and surface coating. Post‐processing methods such as laser ablation, soft lithography, shadow masking, or selective spraying are therefore employed to achieve localized deposition or removal, enabling functional patterning for electrodes and sensing layers in biodegradable devices.^[^ ^241^, ^282^ ^]^

Despite these limitations, solution casting and spray coating remain versatile for integrating biodegradable materials into functional devices. For example, solution casting has been used in successfully fabricating modified PLA with nanochitosan and stearic acid, achieving a water contact angle of 138°, suitable for superhydrophobic applications.^[^ ^297^ ^]^ Similarly, spray coating has proven effective in producing uniform films on non‐planar surfaces, such as ZnO‐coated optical fiber sensors, achieving high sensitivity for acetone vapor detection.^[^ ^298^ ^]^

Advancements in these methods are essential to address their environmental and technical drawbacks. Solution casting's reliance on solvents can be mitigated by transitioning to bio‐based solvents and incorporating solvent recovery systems to minimize pollution. Furthermore, optimizing spray parameters and transitioning to low‐VOC formulations could minimize emissions and improve sustainability. To ensure the precision and uniformity of the fabricated layers, real‐time monitoring systems, such as in situ optical coherence tomography and infrared thermography, can optimize spray processes by detecting defects and adjusting parameters dynamically.^[^ ^299^ ^]^ Hybrid approaches integrating solution casting with techniques like laser ablation and plasma treatment improve adhesion, surface morphology, and scalability.^[^ ^300^ ^]^

### 6.7 Electrospinning

Electrospinning is a highly versatile technique for fabricating biodegradable devices, providing precise control over material structure and properties. It enables the production of ultrafine fibers with tailored porosity and morphology, which are particularly advantageous for environmental applications such as gas and humidity sensing. The use of biodegradable polymers and non‐toxic solvents further reduces environmental impact, making it a more sustainable fabrication method.^[^ ^301^ ^]^ Conductive polymers such as polypyrrole (PPy) and polyaniline (PANI) have been widely used in electrospinning but are not biodegradable.^[^ ^302^, ^303^ ^]^ Electrospun ZnO fibers have been developed for humidity sensing, utilizing their high surface area and nanoporosity for effective moisture adsorption. These fibers displayed excellent sensitivity to varying RH levels, making them promising for environmental monitoring applications.^[^ ^304^ ^]^ However, this method faces challenges, including complex process optimization, difficulties in scaling up production, and the need for precise control of fiber morphology to ensure uniformity. Additionally, post‐processing treatments like NaOH introduce chemical waste and increase production costs, limiting its scalability as a sustainable method.^[^ ^305^ ^]^

The biodegradable electrospun cellulose acetate materials used for VOC sensing, as shown in Figure 9h, reach surface areas of 11.85 m^2^ g^−1^, which is comparable to conventional electrospun nanocomposites containing functionalized additives.^[^ ^44^ ^]^ This surface area enables efficient gas adsorption, making the material suitable for detecting moderate concentrations of VOCs. In comparison, advanced electrospinning techniques that integrate metal oxide nanoparticles can produce surface areas exceeding 100 m^2^ g^−1^, resulting in significantly greater adsorption capacity and improved sensitivity for detecting low concentration VOCs.^[^ ^306^ ^]^

The development of more sustainable solvents and self‐assembly techniques could reduce or eliminate the need for post‐processing treatments—such as thermal curing, chemical rinsing, or purification—which are often required to improve material stability, uniformity, or functionality. Minimizing these additional steps would not only enhance the scalability of the fabrication process but also lower energy consumption and reduce hazardous waste generation.^[^ ^307^ ^]^ Incorporating smart electrospinning strategies, such as artificial intelligence (AI)‐driven process optimization and stimuli‐responsive nanofibers, could enable more autonomous systems capable of real‐time adaptation to environmental changes.

### 6.8 Dip Coating

Dip coating is a versatile and cost‐effective method for fabricating biodegradable device components, including electrodes and encapsulation layers.^[^ ^88^ ^]^ It involves immersing a biodegradable substrate, such as cellulose or PLA, into a solution containing electrode materials like degradable metal particles, followed by controlled withdrawal to achieve uniform deposition.^[^ ^308^ ^]^ This process is particularly suitable for creating thin, conformal electrode layers on substrates with complex geometries, making it ideal for biomedical and environmental applications.^[^ ^184^, ^308^ ^]^ However, challenges remain, including controlling film thickness and uniformity—particularly for intricate substrates—and addressing potential defects like pinholes or cracks that may affect performance. The simplicity and scalability of the method, though, make it an essential tool for sustainable manufacturing. The dip‐coating method employed by Lee et al.,^[^ ^184^ ^]^ as shown in Figure 9i, exemplifies these strengths, offering a straightforward and scalable approach for fabricating cellulose‐based electrodes functionalized with non‐biodegradable MWCNT and metal dichalcogenides.^[^ ^184^ ^]^ Critical issues such as uneven coating due to inconsistent dipping or drying and potential delamination under operational emphasizes the need for further improvements.

The performance of dip‐coating is moderate, as shown by semi‐degradable cellulose‐MXene hybrid electrodes reaching conductivity values of up to 1.91 S cm^−1^.^[^ ^309^ ^]^ In comparison, advanced techniques like vacuum‐assisted filtration and spray coating can achieve significantly higher conductivity and uniformity due to their precise control over material deposition.^[^ ^310^ ^]^ However, these methods involve more complex setups and increased costs, making them less accessible for large‐scale production.

Improving the precision of material deposition through optimized dipping parameters and advanced control systems offers a valuable pathway for enhancing the dip‐coating process. Automation could further enhance uniformity and reproducibility, minimizing operational inconsistencies.

## 7 Powering Strategies for Biodegradable Electronics Dedicated to Environmental Applications

The development of transient systems for environmental applications necessitates power sources that are compatible with biodegradable materials and capable of degrading without harmful residues. These power sources must integrate seamlessly with read‐out, control, and communication electronics, which manage signal acquisition, data processing, and wireless transmission, as previously discussed at the end of the sensor application section. However, identifying and integrating such power solutions remains a significant challenge.

First, achieving sufficient energy density and longevity is problematic, as biodegradable materials used in batteries, supercapacitors, fuel cells, piezoelectric generators, or TENGs often lack the performance and durability of traditional counterparts, leading to shorter lifespans and reduced efficiency.^[^ ^311^ ^]^ This trade‐off makes it challenging to balance environmental benefits with reliable performance.^[^ ^312^ ^]^ Second, these materials must maintain stability under varying environmental conditions, such as temperature fluctuations, RH, and exposure to soil or water, where they are prone to premature degradation.^[^ ^313^ ^]^ Third, ensuring all power source components decompose completely without leaving harmful residues requires meticulous material selection and design.

In this section, various powering strategies suitable for biodegradable devices in environmental applications are described, following a logical sequence: batteries, supercapacitors, RF remote powering and harvesting, photovoltaics, and triboelectric generators.

### 7.1 Batteries

**Figure 10:**
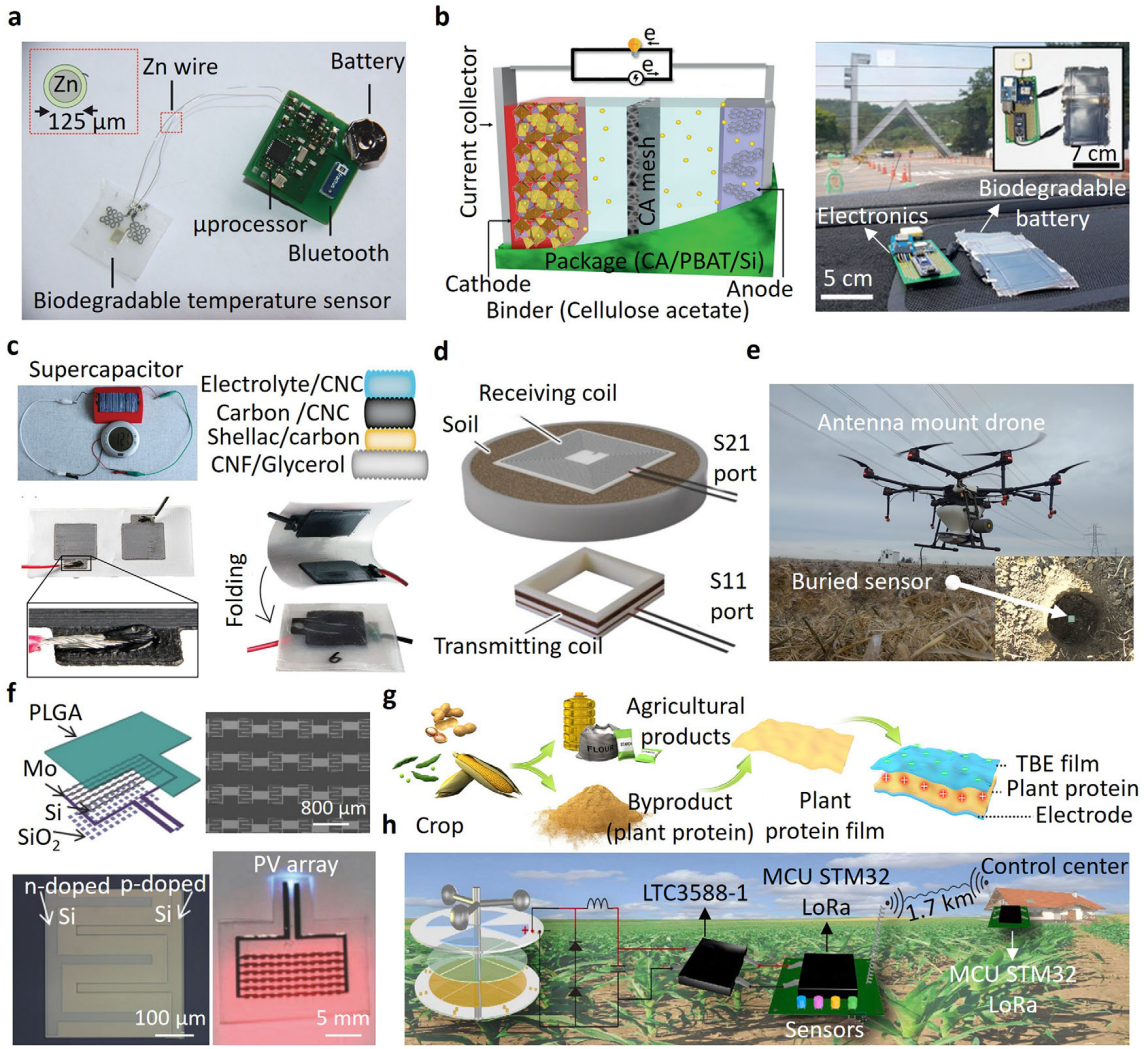
Powering methods for degradable devices: a) A partially biodegradable temperature sensor connected to the electronics with an on‐board battery as power supply. Reproduced with permission.^[^ ^42^ ^]^ Copyright 2017, Wiley. b) Illustration of partially biodegradable battery and its application in supplying power to a board with a GPS module. Reproduced with permission.^[^ ^314^ ^]^ Copyright 2021, Wiley. c) Fully printed biodegradable supercapacitor with the capability to power up an alarm clock. Reproduced with permission.^[^ ^325^ ^]^ Copyright 2021, Wiley. d) Wireless RF powering of a passive biodegradable soil moisture sensor by transmitting coil. Reproduced with permission.^[^ ^74^ ^]^ Copyright 2023, Wiley. e) Photo of the drone‐mounted short‐range antenna reader hovering over a sensor tag placed at a depth of 5 cm for wireless data transmission. Reproduced with permission.^[^ ^32^ ^]^ Copyright 2022, Springer Nature. f) Illustration of a degradable photovoltaic array microfabricated based on doped Si to supply the energy for powering LEDs. Reproduced with permission.^[^ ^334^ ^]^ Copyright 2018, Wiley. g) TENG based on biodegradable plant and protein based triboelectric (TBE) film. Reproduced with permission.^[^ ^335^ ^]^ Copyright 2022, Elsevier. h) Partially biodegradable TENG‐based self‐powered sensing platform for agricultural monitoring. Reproduced with permission.^[^ ^41^ ^]^ Copyright 2022, Elsevier.

Batteries generate energy by driving a chemical reaction between two electrodes immersed in an electrolyte, causing electrons to flow through an external circuit and produce an electrical current that powers connected devices. As shown in **Figure** **10**a, Salvatore et al.^[^ ^42^ ^]^ developed a resistive degradable temperature sensor powered by a Zn‐air coin battery, which provided a compact form factor (7.9 mm diameter) and an energy capacity of 180 mAh at 1.45 V. This design offers adequate computational power with low energy consumption for sensor applications. However, non‐biodegradable components like the active circuit and Zn‐air battery reduce environmental sustainability, as Zn‐air batteries, though efficient, contain non‐biodegradable materials. To overcome this, fully biodegradable batteries, such as sodium‐ion batteries with biodegradable electrodes, separators, electrolytes, and encapsulation, have been developed, breaking down through fungal and hydrolytic degradation.^[^ ^314^ ^]^

Transitioning to biodegradable batteries introduces both challenges and opportunities. As depicted in Figure 10b, Lee et al.^[^ ^314^ ^]^ developed a biodegradable battery system with conductive cellulose‐based electrodes, a propylene carbonate electrolyte, and cellulose‐poly(butylene adipate‐co‐terephthalate) (PBAT) packaging, reducing reliance on synthetic materials and promoting sustainability. It achieves a specific capacity of ≈50 mAh g^−1^ with a 3 V discharge plateau, though this is significantly lower than conventional lithium‐ion batteries (>150 mAh g^−1^), limiting its suitability for high‐energy applications like Global Positioning System (GPS) modules.^[^ ^315^ ^]^ Additionally, the use of propylene carbonate, which is not inherently biodegradable, limits the system's ability to significantly reduce environmental impact. Batteries become more sustainable when they incorporate biodegradable materials, such as starch‐based polymer separators and water‐based electrolytes. Using non‐toxic, renewable electrodes derived from lignin or algae further enhance their sustainability. These components are manufactured through low‐energy processes that conserve resources and reduce environmental harm.^[^ ^316^, ^317^ ^]^

Paper‐based Zn–air batteries have achieved power densities as high as 102 mW cm^−2^ by improving cathode structures for better oxygen reduction reaction (ORR) performance.^[^ ^318^ ^]^ While these results are promising, their energy output still lags behind that of conventional non‐biodegradable batteries. Similarly, flexible Zn‐air batteries integrated with strain sensors have achieved power densities of 82.5 mW cm^−2^ and maintained consistent performance under repeated bending, stretching, and compression. This mechanical resilience makes them highly suitable for on‐animal sensing collars used in biodiversity monitoring, where devices must endure continuous movement and deformation.^[^ ^319^, ^320^ ^]^

Advances in materials science offer promising solutions to these challenges. Hydrogel‐based electrolytes, for example, have achieved energy efficiencies exceeding 73%, along with enhanced stability across diverse operating conditions.^[^ ^321^ ^]^ Future directions involve replacing the propylene carbonate electrolyte in biodegradable batteries with fully biodegradable alternatives, such as hydrogel‐based or ionic liquid formulations. Additionally, enhancing the energy density of cellulose‐based electrodes by incorporating advanced biopolymer composites or nanostructured materials could improve capacity and power output.

Biodegradable batteries still fall significantly behind state‐of‐the‐art technologies like lithium‐ion batteries regarding energy density, capacity, and lifespan. For example, while lithium‐ion batteries routinely achieve 150–250 mAh g^−1^ capacities, biodegradable batteries often remain below 100 mAh g^−1^, limiting their utility in high‐demand applications like Internet of Things (IoT) devices. However, biodegradable batteries are already promising in low‐power applications such as environmental sensors, where capacities and lifespans of weeks to months could suffice.^[^ ^322^ ^]^ Improvements in energy density, scalability, and material stability are essential to reach real‐world use in IoT devices. Advances in nanostructured biopolymer electrodes, biodegradable solid‐state electrolytes, and hybrid energy storage systems will be crucial in bridging the performance gap between biodegradable and conventional battery systems.

### 7.2 Supercapacitors

Supercapacitors store energy electrostatically through the separation of charge in an electric double layer, unlike batteries, which store energy chemically.^[^ ^323^ ^]^ The key advantages of supercapacitors include rapid charge and discharge cycles, high power density, long cycle life, and minimal degradation over time.^[^ ^323^ ^]^ However, they are limited by lower energy density compared to batteries, higher self‐discharge rates, and higher costs, which restrict their use to applications requiring quick bursts of power rather than long‐term energy storage.^[^ ^324^ ^]^

The fully direct ink‐printed biodegradable supercapacitor developed by Aebey et al.^[^ ^325^ ^]^ (see Figure 10c) represents a significant advancement in sustainable energy storage. Leveraging nanocellulose for rheological modification and structural integrity, carbon for high‐surface‐area electrodes and conductivity, and glycerol as a dual‐function plasticizer and electrolyte solvent, this design achieved a capacitance of 25.6 F g^−1^, which is ten times higher than that of previously reported fully printed devices.^[^ ^326^, ^327^ ^]^ However, the operational time—powering an alarm clock for just over 20 min following a 3 min charge—highlights its limited energy density for extended use in practical applications.

Biodegradable supercapacitors exhibit lower energy density and durability compared to conventional systems. For instance, printed supercapacitors integrating activated carbon inks exhibits energy densities exceeding 127.8 mF cm^−2^ due to optimized electrode materials and scalable direct ink writing techniques.^[^ ^328^ ^]^ Future advancements could involve replacing glycerol with bio‐based ionic liquids or hydrogel electrolytes to improve both biodegradability and electrolyte performance. Incorporating materials like lignin‐based carbon composites or cellulose‐nanofiber‐reinforced inks may further enhance energy density and durability.^[^ ^329^ ^]^ Using biodegradable carbon sources, such as coconut shells or algae, paired with water‐based electrolytes, could minimize toxic waste while offering sustainable and efficient energy storage. These materials can form nanoporous carbon electrodes with high surface area, enabling excellent capacitance and energy density for next‐generation biodegradable supercapacitors.^[^ ^330^ ^]^

### 7.3 RF Energy Harvesting and Remote Powering

The use of RF‐based energy transfer for biodegradable devices represents a major advancement in ecosystem and environmental monitoring, enabling wireless powering and data transmission for remote sensing applications. This approach harnesses electromagnetic spanning frequencies from the kHz range to tens of GHz to actively transmit or passively harvest power. As a result, biodegradable devices can operate autonomously while minimizing their environmental impact.^[^ ^32^ ^]^

RF remote powering involves the active transmission of electromagnetic energy from an external source to a receiver integrated within the biodegradable device. RF‐based powering is particularly advantageous for long‐range applications, supporting reliable energy delivery to biodegradable sensors and monitoring devices in remote or inaccessible areas; power transmission has been demonstrated over distances of 10 m,^[^ ^331^ ^]^ 4m,^[^ ^332^ ^]^ and 1.54 km.^[^ ^333^ ^]^ However, it faces challenges such as signal attenuation through foliage over distances, necessitating innovative solutions to ensure consistent performance in complex electromagnetic wave propagation environments.^[^ ^25^ ^]^

In contrast, RF energy harvesting relies on passive collection of ambient electromagnetic energy from existing sources such as Wi‐Fi or Global System for Mobile Communications (GSM) signals. Although it provides much lower power levels (typically in the µW to mW range), it enables battery‐free operation for ultra‐low‐power biodegradable sensors deployed in dense or hard‐to‐reach environments.

Wireless sensors in complex environments rely on highly efficient antenna designs for power harvesting and data transmission (or backscattering).^[^ ^336^ ^]^ The antenna plays a critical role in determining the overall system's reading range, signal‐to‐noise ratio, and power efficiency. Key challenges in antenna design include: 1) the antenna radiation efficiency‐radiation efficiency refers to the proportion of power delivered to the antenna that is successfully radiated as electromagnetic waves, rather than being lost as heat. This efficiency is often reduced by ohmic losses in the conductive materials (due to electrical resistance) and dielectric losses in the substrate materials (due to energy dissipation within the dielectric). Minimizing these losses through appropriate material selection and advanced design techniques is critical to improving overall antenna performance;^[^ ^337^ ^]^ 2) miniaturization‐designing compact antennas is particularly difficult at sub‐GHz frequencies, where the long wavelengths require physically large antenna structures for efficient operation. This size requirement makes it difficult to fit antennas into small devices without significantly reducing their efficiency or overall performance; and 3) impedance robustness‐maintaining a stable input impedance is essential for efficient power transfer between an antenna and its associated transmitter or receiver. However, in real‐world conditions, nearby materials—such as the human body, water, or soil—can interact with the antenna's electromagnetic fields. These interactions can shift the antenna's impedance away from its intended value, a phenomenon known as detuning, which reduces efficiency and degrades performance. Robust antenna designs must therefore be resilient to such environmental influences and maintain stable impedance under varying operating conditions.

As depicted in Figure 10d, the degradable and wireless soil moisture sensor was fabricated by Kasuga et al.,^[^ ^74^ ^]^ employs short‐range electromagnetic induction to wirelessly transfer power into a coil laminated onto a cellulosic substrate. The required power is supplied by an external non‐biodegradable coil positioned ≈10 cm away. However, environmental factors affect energy transmission efficiency; for instance, a 40% increase in soil moisture led to a 3% reduction in efficiency. This highlights the challenge of maintaining consistent performance under varying soil conditions, such as changes in conductivity and moisture content.^[^ ^74^ ^]^

Compared to conventional systems, wireless power transfer through the soil using electromagnetic induction has shown robust capabilities. A similar inductively coupled system achieved 20 mW of power transfer through 15 cm of soil at a 31–46% moisture range, maintaining efficiency in different moisture conditions.^[^ ^338^ ^]^

The drone‐mounted antenna system (see Figure 10e) achieved a greater powering distance of 25 cm but required precise sensor orientation and optimal conditions for effective transmission. Other studies have shown that power transmission distances can reach up to 30 cm using optimized inductive techniques; however, more challenging soil environments—such as those with high clay content, elevated moisture levels, or high salinity—can significantly increase dielectric losses and reduce transmission efficiency. These soil characteristics attenuate electromagnetic signals more severely, leading to increased power losses and reduced effective range.^[^ ^339^ ^]^

One of the significant challenges for these systems is the impact of soil medium properties, such as moisture content, on energy transmission efficiency. As observed, a slight increase in moisture content can reduce transmission efficiency, which complicates the use of such systems in varied environmental conditions. Advances such as the use of ferrite materials to enhance the inductance of coils have been shown to improve efficiency and minimize power losses in soils with higher conductivity.^[^ ^340^ ^]^

Biodegradable RF systems are advancing through the use of cellulose‐based materials for antennas and rectifiers.^[^ ^341^ ^]^ Antenna efficiency can be improved through advanced designs, such as metasurface integration, which enhance energy harvesting and minimize signal losses in challenging environments like soil or dense vegetation.^[^ ^342^ ^]^ Operating frequencies are optimized to reduce attenuation while maintaining compatibility with biodegradable materials, ensuring reliable power delivery.^[^ ^343^ ^]^ Despite lower efficiencies (5–15%) compared to conventional systems (>80%),^[^ ^344^, ^345^ ^]^ they offer sustainable solutions for applications in sensitive environments, such as agricultural fields or protected ecosystems.

### 7.4 Photovoltaic Energy Harvesting

Photovoltaic energy harvesting converts sunlight into electricity using semiconductors —most commonly Si—which generate electrical current when exposed to photons.^[^ ^334^, ^346^ ^]^ Degradable photovoltaic devices aim to harness solar energy while reducing greenhouse gas emissions and environmental impact.^[^ ^347^ ^]^ While conventional Si can be used in thin, and degradable formats without significantly compromising performance, many degradable alternatives rely on organic or composite materials with inherently lower energy conversion efficiencies. As a result, larger surface areas are often needed to achieve comparable power output, which can be a limitation in space‐constrained environments such as forest monitoring stations, underwater sensing platforms, or soil‐embedded agricultural sensors.^[^ ^348^, ^349^ ^]^

Lu et al.^[^ ^334^ ^]^ a degradable Si‐based photovoltaic microcell array (≈100 µm thick), designed primarily for implantable biomedical powering, as shown in Figure 10f. The device demonstrated degradation in an aqueous environment and delivered a power output of ≈122 µW under 1 sun illumination with an open‐circuit voltage (Voc) of 4.84 V. However, its operational output of 64 µW limits its applicability to low‐power devices.^[^ ^334^ ^]^ Non‐biodegradable micro‐concentrator photovoltaic systems using multijunction solar cells have reached efficiencies of 33.8% under concentrated sunlight, far surpassing the performance of biodegradable arrays.^[^ ^350^ ^]^ Flexible non‐biodegradable organic photovoltaics (OPVs) have also shown promise, with specific power outputs of 0.38 W/g and durability over 5000 bending cycles, highlighting their potential for on‐animal monitoring systems.^[^ ^351^ ^]^

Future improvements could focus on enhancing the efficiency and durability of degradable photovoltaics by engineering advanced hybrid materials, such as biodegradable organic semiconductors, to improve light absorption, charge transport, and overall energy conversion.^[^ ^352^, ^353^, ^354^ ^]^ This can be realized through the rational design of degradable semiconductors with fine‐tuned bandgaps, high carrier mobility, and durability in real‐world environmental conditions.

### 7.5 Triboelectric Nanogenerators

Triboelectric nanogenerators exploit environmental mechanical energy via the triboelectric effect and electrostatic induction to produce electricity. This method entails contacting and separating materials with differing electronegativities, generating a potential difference that facilitates electron movement.^[^ ^355^ ^]^ TENGs are ideal for biodegradable environmental monitoring devices, as they generate energy from mechanical sources like wind, vibrations, and human motion. Their lightweight, flexible design allows integration into portable systems for remote tracking, such as paper‐based TENGs used for energy harvesting and real‐time sensing in ecosystems lacking conventional power sources.^[^ ^356^ ^]^ Molecularly doped biodegradable TENGs have shown improved performance, making them suitable for dynamic sensing applications such as respiratory monitoring.^[^ ^357^ ^]^ Despite these advantages, challenges such as low energy output and durability limit their performance in applications requiring continuous operation and high power consumption, such as long‐term environmental monitoring, real‐time data transmission, and large‐scale sensor networks.^[^ ^358^ ^]^

As shown in Figure 10g, the use of plant‐based proteins, such as rice protein, peanut protein isolate, soybean protein isolate, wheat gluten, and zein, as triboelectric materials highlight the potential of biodegradable TENGs for sustainable energy harvesting.^[^ ^335^ ^]^ Biodegradable materials, such as chitosan (from crustacean shells), cellulose (from plants), and food waste, further expand the range of sustainable options for TENG fabrication.^[^ ^359^, ^360^ ^]^ However, these materials often exhibit variability in triboelectric charge separation and surface interactions over time, leading to inconsistent performance.^[^ ^335^, ^361^ ^]^ Environmental factors such as humidity and UV exposure contribute to these issues, accelerating material degradation and reducing device efficiency.^[^ ^357^ ^]^ Despite these challenges, progress has been made in developing functional biodegradable energy harvesters. Biodegradable TENGs using compostable cellulose‐poly(butylene succinate) composites have achieved power densities of 143 mW m^−2^, which, although competitive, still lag behind non‐biodegradable alternatives.^[^ ^362^ ^]^

The corn husk‐based TENG developed by Gu et al.^[^ ^41^ ^]^ (Figure 10h) provides an example of leveraging agricultural by‐products for energy harvesting. Achieving an output voltage of 3.2 kV and a transferred charge of 300 nC, it highlights the feasibility of using biodegradable materials for powering sensors in agricultural settings. However, the energy density and power retention remain less competitive than synthetic‐based TENGs. In comparison, sunflower husk TENGs have achieved higher power outputs of 1200 µW under specific conditions, outperforming corn husk‐based systems.^[^ ^363^ ^]^ Recent studies have shown that biodegradable supercapacitors integrated with TENGs can achieve energy densities of up to 5.5 mWh cm^−2^, sufficient to power low‐energy devices like temperature and humidity sensors in remote agricultural fields.^[^ ^362^ ^]^ Integrating supercapacitors into TENG designs addresses energy intermittency, enabling consistent power output. Supercapacitors, with their rapid charge–discharge cycles and high power density, store harvested energy for continuous use, supporting long‐term environmental monitoring.^[^ ^364^ ^]^ Enhancing material durability and triboelectric efficiency are essential to address these limitations. Doping biodegradable materials with additives like poly(propylene glycol) or ethyl cellulose improves charge density and mechanical stability.^[^ ^365^ ^]^ **Table** **5** summarizes the powering strategies covered in this chapter with their mechanisms and key performance metrics.

**Table 5:** Powering strategies for degradable electronics dedicated to environmental applications.

Powering strategy	Mechanism	Performance highlights	Refs.
Batteries	Redox reactions between electrodes and electrolytes	Specific capacity ≈50 mAh g^−1^ at 3 V; Power density up to 102 mW cm^−2^	[42, 314, 322]
RF Energy Harvesting	Electromagnetic induction	Wireless powering up to 1.54 km; Through‐soil transfer: 20 mW at 15 cm depth	[25, 74, 331]
Supercapacitors	Charge storage via electric double‐layer and pseudocapacitance	Capacitance: 25.6 F g^−1^; Energy density >127.8 mF cm^−2^	[325, 326, 327]
Photovoltaics	Conversion of photon energy into electricity via photovoltaic effect	Power output ≈122 µW at 1 sun exposure (Voc 4.84 V)	[334, 346, 347, 351]
TENGs	Triboelectric effect combined with electrostatic induction	Output voltage: 3.2 kV, transferred charge 300 nC; Power output up to 1200 µW; Hybrid TENG‐supercapacitor systems: 5.5 mWh cm^−2^	[41, 335, 365]

## 8 Conclusion and Perspective

Advancements in conventional satellites, sensors, and monitoring systems have enhanced agricultural productivity and ecosystem management; however, they are constrained by limitations such as spatial constraints and environmental impact. Ecoresorbable electronics offer a sustainable solution, enabling large‐scale, temporary ecological and environmental monitoring while naturally degrading after their operation.

A variety of biodegradable materials, including inorganics, synthetic, and natural polymers, have been utilized so far in transient devices. Biopolymers such as PLA, PVA, PHBV, and Ecoflex are also widely employed for their flexibility, processability, and tunable degradation rates. Cellulose, wood, and silk are commonly chosen for their degradability and cost‐effectiveness, though their inconsistencies in properties pose challenges for large‐scale applications. Natural‐derived materials such as lignin, tannins, and gelatin present promising alternatives for different device components, expanding the material choices for future biodegradable electronics.

Despite these advancements, many sensors remain only partially biodegradable due to poor conductivity, limited electrochemical activity, or structural instability of degradable electrodes. Further research is required to improve their performance by enhancing conductivity through doped biopolymers,^[^ ^366^ ^]^ increasing electrochemical activity with 2D nanostructured coatings,^[^ ^367^ ^]^ and improving structural stability using cross‐linked natural polymers.^[^ ^368^ ^]^ Encapsulation critically governs both the operational lifetime and degradation kinetics of biodegradable sensors. Device longevity can be precisely controlled by tuning encapsulation thickness, polymer cross‐linking density, or compositional gradients that regulate permeability to water and oxygen. Ecoflex coatings provide effective short‐term moisture barriers, while multilayer Ecoflex/oxide stacks enhance durability in saline and humid environments.^[^ ^369^, ^370^ ^]^ The morphology and packing density of secondary particles within encapsulant composites further influence dielectric properties and diffusivity, enabling controlled degradation without loss of analytical performance.^[^ ^370^ ^]^ For EC sensors, encapsulation remains particularly challenging because the functionalized electrode surface must remain accessible to target ions and protons, limiting the use of fully impermeable barriers. Future designs should focus on integrating proton‐conductive yet low‐swelling biodegradable encapsulants that preserve electrochemical accessibility while mitigating moisture‐induced drift. For temperature sensors, shellac encapsulation provides humidity‐insensitive protection, maintaining measurement reliability over extended exposure.^[^ ^43^ ^]^ Cellulose‐nanofiber layers serve as optically transparent and moisture‐resistant encapsulants for photodetectors in outdoor monitoring.^[^ ^371^ ^]^ Gradient encapsulation architectures with spatially tuned permeability present an effective route to synchronize degradation with functional lifetime, providing tailored stability across soil, air, and aquatic environments.^[^ ^372^ ^]^ Collectively, these strategies establish a practical framework for adjusting sensor lifespan to match specific monitoring durations, ensuring reliable operation, predictable degradation, and environmental safety.

Moreover, the integration of LCA frameworks into the design and optimization of biodegradable electronics is critical for quantifying their net environmental performance relative to conventional non‐degradable counterparts. LCA‐informed material and process engineering enables systematic evaluation of cradle‐to‐grave impacts, facilitating the reduction of embodied carbon, cumulative energy demand, resource depletion, and end‐of‐life waste generation. Such a data‐driven approach ensures that the environmental benefits of biodegradability are realized holistically across the full device lifecycle, from raw material extraction to final degradation.

The degradation behavior of biodegradable electronics varies widely depending on environmental conditions. While mechanisms such as fungal‐assisted degradation and hydrolysis have been studied, reported degradation rates are often inconsistent due to non‐standardized testing environments across soil, air, and water. To enable meaningful comparisons, standardized measurement conditions—such as temperature, humidity, light intensity, and pollutant concentration—must be established. The European Committee for Standardization (CEN) and the Joint Research Center (JRC) of the European Commission, both responsible for water and soil quality monitoring, could serve as key institutions in establishing biodegradability‐specific metrics, helping to ensure consistency and repeatability across studies.

Currently, biodegradable devices are primarily used for environmental monitoring and agriculture, contributing to optimized crop growth and productivity. While these devices have been tested for tracking environmental parameters in air, water, and soil, most studies are restricted to controlled laboratory settings, lacking exposure to actual environmental dynamics. Limited field testing hinders their validation under natural conditions, creating a gap between laboratory performance and real‐world applicability. Additionally, their potential for monitoring other ecosystems—such as forests, oceans, and mountains—remains largely unexplored. In these environments, biodegradable devices could play a vital role in conservation, biodiversity assessments, climate change research, and scientific discoveries by providing real‐time data on habitat health, species distribution, and environmental changes in remote and sensitive regions. However, in general, the performance of fully biodegradable devices is inferior to that of non‐ or partially biodegradable alternatives, limiting their performance in practical applications. Therefore, there is an urgent need for further research and technological advancements to enhance the reliability, sensitivity, and overall functionality of fully biodegradable devices, ensuring they function effectively in real‐world applications. Expanding their application to these ecosystems with improved performance could enhance ecological research, support early‐warning systems for environmental degradation, and promote sustainable resource management.

The selection of a fabrication method compatible with the chosen materials is a critical step in developing transient devices. Techniques such as screen printing, spray/dip coating, and 3D printing have been widely utilized due to their cost‐effectiveness and simplicity. However, scalability remains a key challenge for these methods. Cleanroom‐based microfabrication enables batch fabrication and facilitates the integration of biodegradable materials into complex electronic systems. Laser‐based microfabrication, while a serial process, offers high precision and flexibility, making it suitable for detailed structuring and prototyping of biodegradable components. Industrial applications, particularly in large‐scale agricultural and environmental monitoring, benefit from roll‐to‐roll (R2R) printing, which offers a scalable and cost‐effective approach for continuous fabrication of biodegradable devices. Its high‐throughput capability enables large‐area production while maintaining uniform film morphology and consistent electrical performance through precise control of process parameters such as ink rheology, drying rate, web tension, and substrate adhesion. This makes R2R printing a key technology for advancing biodegradable electronics from laboratory research to industrial manufacturing.

Sensing and data communication remain critical aspects in advancing (bio)degradable devices for environmental and ecological monitoring. In fully transient systems, sensing is typically achieved through passive resonant elements configured as chipless RFID tags, which enable wireless interrogation without the need for integrated microchips or batteries. In these systems, data transfer occurs via backscattering communication, where a remote station transmits a carrier signal that is modulated and reflected by (bio)degradable coils or antennas integrated within the sensor. This configuration enables autonomous, battery‐free operation, making it ideal for short‐term deployments in remote or sensitive ecosystems. Flexible and conformal antennas are particularly essential in devices for environmental monitoring attached to irregular surfaces (e.g., soil, leaves, or rocks), where the design of conformal antennas and arrays critically determines radiation efficiency, coupling stability, and communication reliability.^[^ ^373^ ^]^ Hybrid systems, by contrast, combine (bio)degradable sensors with reusable or recyclable electronic modules to achieve higher signal fidelity, extended communication range, and real‐time data management. CMOS‐based front‐end circuits perform excitation, measurement, and processing, while wireless modules such as BLE, Wi‐Fi, or LoRa transceivers facilitate telemetry and cloud integration. Multiplexed readout architectures further improve scalability and power efficiency, and the emerging development of (bio)degradable CMOS technologies promises to unify sensing, processing, and communication functions within transient platforms. Coupled with unmanned aerial vehicle (UAV)‐based relay networks for data retrieval, these advances can enable robust, energy‐efficient, and autonomous ecological monitoring systems operating over large spatial and temporal scales.

Wireless powering is an equally significant challenge, as most biodegradable devices still rely on wired setups that are impractical for remote or inaccessible environments. To overcome this, biodegradable power sources such as batteries, supercapacitors, RF remote powering systems, photovoltaics, and TENGs are being explored. Among these, RF remote powering, which involves the active transmission of energy from an external source, is particularly promising for enabling long‐range wireless operation. On the other hand, RF energy harvesting, which passively receives ambient electromagnetic energy, is better suited for low‐power battery‐free operation. While only a few fully passive systems composed entirely of biodegradable components have been demonstrated, their operational range could be extended to hundreds of meters through i) high‐Q biodegradable resonant structures to maximize coupling efficiency,^[^ ^374^ ^]^ ii) Optimization of operating frequency in complex and lossy environments to balance electromagnetic attenuation and antenna gain,^[^ ^375^ ^]^ iii) biodegradable antennas with large effective apertures and minimal electromagnetic losses to improve both radiation efficiency and power transfer, and iv) metasurface‐assisted designs that concentrate and redirect incident electromagnetic fields to enhance power transfer. Integrating these powering approaches with the communication and signal‐processing solutions discussed above will be crucial for realizing autonomous, large‐scale biodegradable sensor networks capable of continuous environmental monitoring with minimal ecological impact.

## Conflict of Interest

The authors declare no conflict of interest.
